# Biomechanical Sensing Using Gas Bubbles Oscillations in Liquids and Adjacent Technologies: Theory and Practical Applications

**DOI:** 10.3390/bios12080624

**Published:** 2022-08-10

**Authors:** Ivan S. Maksymov, Bui Quoc Huy Nguyen, Sergey A. Suslov

**Affiliations:** 1 Optical Sciences Centre, Swinburne University of Technology, Hawthorn, VIC 3122, Australia; 2 Department of Mathematics, Swinburne University of Technology, Hawthorn, VIC 3122, Australia

**Keywords:** biosensing, biomechanics, cellular viscoelasticity, vibrations, nonlinear acoustics, acousto-optics, bubbles, acoustic frequency combs, artificial intelligence, physics-informed neural networks

## Abstract

Gas bubbles present in liquids underpin many natural phenomena and human-developed technologies that improve the quality of life. Since all living organisms are predominantly made of water, they may also contain bubbles—introduced both naturally and artificially—that can serve as biomechanical sensors operating in hard-to-reach places inside a living body and emitting signals that can be detected by common equipment used in ultrasound and photoacoustic imaging procedures. This kind of biosensor is the focus of the present article, where we critically review the emergent sensing technologies based on acoustically driven oscillations of bubbles in liquids and bodily fluids. This review is intended for a broad biosensing community and transdisciplinary researchers translating novel ideas from theory to experiment and then to practice. To this end, all discussions in this review are written in a language that is accessible to non-experts in specific fields of acoustics, fluid dynamics and acousto-optics.

## 1. Introduction and Motivation

Gas bubbles in liquids underpin many natural phenomena and human-designed technologies [1,2]. For example, it is well-known that the sound of running water originates from periodic expansions and contractions of spherical bubbles trapped in the bulk of fluid [3,4]. It has also been hypothesised that oscillations and collapse of bubbles in the primordial ocean might have contributed to the origin of life on Earth [5] and that the presence of bubbles in the brain could be associated with blast-induced neurotraumas [6,7] and some debilitating diseases that significantly change lives of millions of people [8]. In turn, artificial bubbles are essential in many modern technologies including drug delivery [9,10,11] and high-contrast medical ultrasound imaging procedures [10,12,13], texture tailoring in food industry [14], natural gas recovery in petroleum industry [15], material synthesis in material sciences [16], lab-on-a-chip devices [17], wastewater treatment systems [18], sonochemistry (enhancement and alternation of chemical reactions by means of ultrasound) [19] and sonoprocessing [19,20] and underwater acoustic communication [21]. The physics of bubbles is also highly relevant to processes taking place during underwater explosions [22].

Thus, due to the natural presence of bubbles in body fluids and tissues [6,7] and the availability of commercial technologies enabling a safe and well-controlled introduction of bubbles into a living body [9,10,11,12], physical properties of bubbles create novel opportunities for biomechanical sensing that underpins the mainstream discussion in this review article. The analysis of biomechanical properties can help differentiate healthy single biological cells and tissues from abnormal ones, thereby serving as a useful tool for biologists and pathologist [23,24]. Mechanical properties of cells are also closely associated with a wide range of cellular responses including cytoskeletal remodelling, synthesis of extracellular matrix proteins and altered expression of genes [25,26,27]. For example, it has been shown that deformation of a cell can result in changes in cellular stiffness, which has been interpreted as an evidence of a nonlinear elastic response of actin cytoskeleton [28,29].

**Figure 1:**
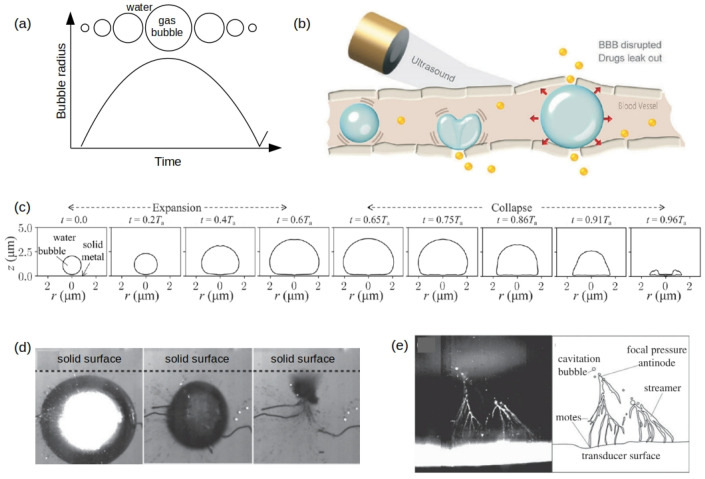
Illustration of bubble oscillation in the bulk of water and near a surface such as the wall of a blood vessel discussed in Section 1. Similar behaviour is observed when a bubble interacts with the wall of an individual biological cell (e.g., [32]). (**a**) Schematic radius-vs-time diagram for a bubble oscillating in the bulk of water. Bubble shapes at different times are shown above the curve during a single oscillation cycle. The pressure inside a bubble is high at the beginning and at the end of the oscillation cycle and is low in the middle. (**b**) Illustration of the principle of drug delivery through the blood-brain barrier (BBB) using bubbles trapped inside a small blood vessel of the brain. Bubble oscillations are driven by an external ultrasound wave transducer and the drug (small spheres in the picture) pass through the wall of a blood vessel when the bubble either reaches its maximum radius or collapses and forms a water jet. Reproduced from [10] under the terms of a Creative Commons license. (**c**) Representative computational axisymmetric profiles of a microbubble during its expansion and collapse near a solid surface. Parameter $T_{a}$ is the period of the sinusoidal acoustic pressure wave with f=1.5 MHz. The pressure wave incident along the *z*-axis towards the surface has the amplitude of 200 kPa. Reproduced from [33] under the terms of a Creative Commons license. (**d**) Experimental observation of a bubble collapse. The first frame shows the bubble at its maximum radius. The bubble shrinks and moves towards the boundary in the second frame and then it collapses forming a jet directed towards the surface. (**e**) Experimental observation of bubble streams that originate mostly from the surface of a transducer and move towards the focal point near the top of the left image. A schematic of a streamer trace (the right image) shows how bubbles emerge from the motes–the source of the streamers. Reproduced with permission of The Royal Society (UK) from [34]. Copyright 2015.

**Figure 2:**
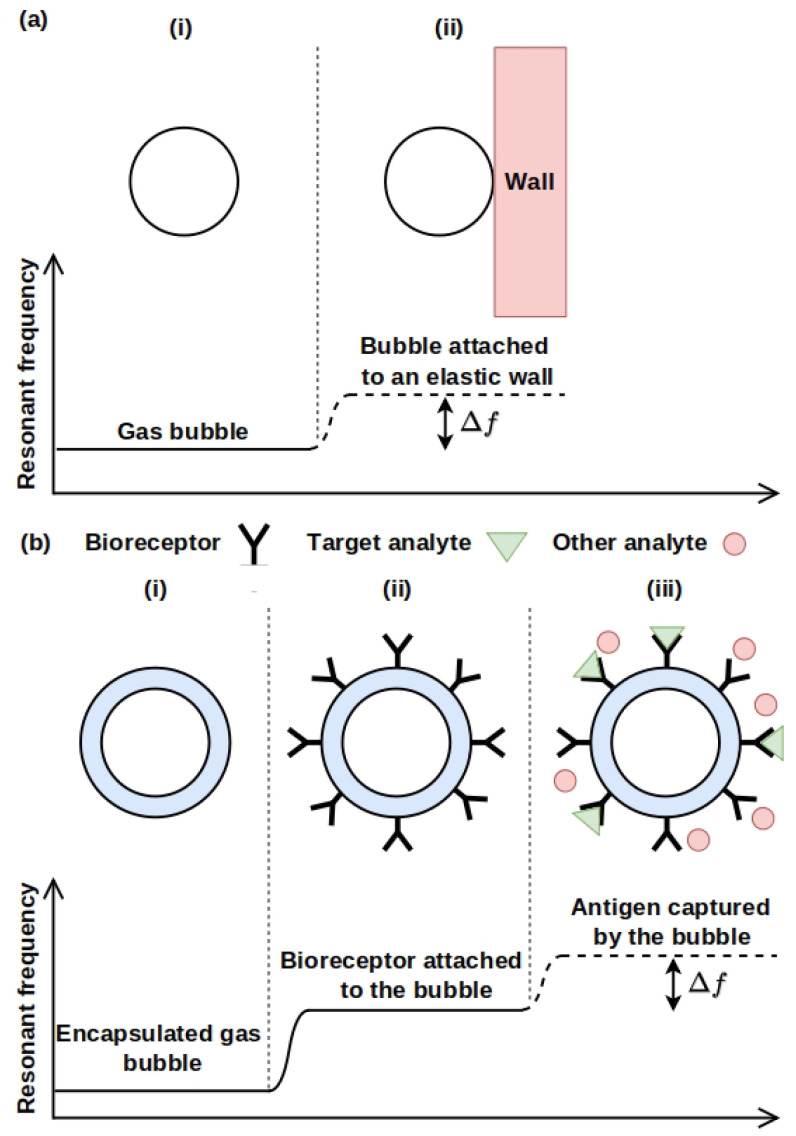
(**a**) Schematic of the operating principle of gas bubble-based sensors for probing mechanical properties of an elastic wall (e.g., a wall of a blood vessel wall or a biological cell). In a practical realisation, microbubble oscillations are driven by ultrasound waves. The resonance frequency shift Δf caused by the approach of the bubble to a wall is detected using a technically simple integrated electronic circuit. At the post-processing step, the measured value of Δf is correlated with the values of material parameters that characterise elastic properties of the wall as discussed in Section 2. (**b**) Illustration of complementary biochemical sensing properties of gas bubble sensors shown in panel (**a**). The resonance frequency *f* of a microbubble (stage (i)) first increases when its surface is functionalised using a biorecognition ligand (e.g., an antibody, stage (ii)). It increases further when the bubble covered by an antibody captures analyte (stage (iii)), thereby resulting in a modification Δf of the resonant frequency that is detected using the same approach as in panel (**a**).

A gas bubble in a body fluid such as blood can periodically expand and collapse (oscillate), see Figure 1a. A force driving such a motion can be created by ultrasound pressure waves produced using equipment employed in commercial ultrasound imaging systems. From the physical point of view, an oscillating bubble can be regarded as a mechanical resonance system located in a hard-to-reach place such as a blood vessel in the brain (Figure 1b). Naturally, the resonance frequency of the oscillating gas bubble and the linewidth of the resonance peak in the frequency spectrum of its response change when the properties of the environment surrounding it are modified [1]. For example, this is the case when a bubble oscillates near a blood vessel wall [30,31] or in the vicinity of a single biological cell that is larger than the equilibrium radius of the bubble. These two scenarios are shown schematically in Figure 2.

Even a very small change Δf in the resonance frequency of a bubble that oscillates near an elastic wall can be detected remotely with high precision by employing ultrasound waves generated by a conventional piezoelectric transducer located outside the body (Figure 2a). Moreover, an ultrasound-based detection avoids any surgical intervention. This is in contrast to imaging and sensing techniques that use lasers inside a living body. Since light is strongly attenuated by biological tissues and fluids [35,36], such techniques require an invasive insertion of an optical fibre guiding the light [37].

Of course, no surgical intervention is needed when the light is used to image individual biological cells and to monitor their physiology, which is, for example, the case with fluorescence-based microscopy [38]. It involves the absorption of light energy (a photon) by fluorophores that enable visualisation of specific biomolecules. Fluorophore molecules or a nanoparticles can be introduced to live specimens directly to highlight the interior of a cell in situ [39]. The light absorption is followed by re-emission of a photon with a lower energy and a longer wavelength. Hence, fluorescence microscopy separates the re-emitted lower intensity light from its excitation counterpart creating an image.

While fluorescence microscopy has become a major tool for monitoring cell physiology, this technique has certain fundamental physical limitations that restrict its capability of revealing mechanical properties of biological cells. Firstly, the operating time of fluorescent molecules and nanoparticles introduced into a cell at the beginning of imaging is often insufficient for real-time monitoring of the cell leading to the physico-chemical effect called photobleaching [39]. This problem cannot be resolved by increasing the intensity of the excitation light since it may cause photodamage [40] to a cell. While novel fluorophores that require less intense excitation light and that are less prone to photobleaching have been developed, their long-term effect on the cell physiology has not been studied in much detail yet. Therefore, their practical use remains very limited [39]. Secondly, fluorescence microscopy alone cannot be used to detect mechanical properties of cells. Thus, it has been combined with other approaches that enable one to mechanically stimulate cells in vitro using, for example, electrically stretchable materials and atomic force microscopy (AFM) [27]. However, despite certain advantages offered by the resulting hybrid technique its experimental implementation is more complicated than a typical fluorescence microscope [38] because (i) the AFM tip can disrupt the cell, (ii) mechanical parts of the AFM setup can obscure the optical path of a fluorescence microscope, (iii) stretching of cells with elastomers requires applying step voltage signals to them with a very large amplitude of about 4000 V and (iv) elastomer films develop a hysteretic response that needs to be taken into account in the analysis of raw data [27]. Finally, the use of fluorescence-based probes during in vitro fertilisation (IVF) poses significant problems. Since their effect on the embryo development is unknown, a direct contact of fluorophores with an embryo is ethically unsound and is not permitted in many regulatory jurisdictions [41].

Many of the aforementioned drawbacks of fluorescence-based and similar imaging and sensing techniques are not expected to exist in bubble-based sensors, which motivates this review article. Indeed, speaking of a potential application in the very important and ethically sensitive field of IVF, it is well-established that both low-intensity ultrasound waves [42] and microbubble ultrasound contrast agents are safe for obstetric imaging applications [43]. Therefore, it is plausible that bubble-based sensors would be suitable in this field. Moreover, in areas, where the use of fluorophores is allowed, bubble-based sensors may be combined with standard fluorescence microscopy techniques. In that case, compared with AFM tips, bubbles should be more biologically compatible and less disruptive to a cell provided that a right operating regime is chosen (see discussion below). In addition, compared with elastomer films used for cell stretching, controlling bubbles would not require the application of potentially unsafe electrical pulses. Yet, an oscillating bubble can provide an extra detection mechanism when it is encapsulated with a biorecognition ligand (Figure 2b) [44,45]. This enhances the capability range of the emergent bubble-based biosensing platform even further.

We outline physical regimes of gas bubble oscillations that should be suitable for sensing purposes discussed in this article next. We also show that oscillating bubbles may be damaging if driven by high-amplitude ultrasound waves and/or are located close to a cell surface.

It often suffices to assume that oscillating bubbles remain spherical (see Figure 1a and Figure 2a) [1,2,46] or quasi-spherical [30,31]. However, a bubble oscillating near a solid surface may collapse non-spherically forming a powerful jet and producing acoustic shock waves [34,47,48], see Figure 1c, and resulting in the formation of a toroidal bubble at the end of the collapse stage. Computations and experimental measurements show that such a jet impinges a solid surface creating pressure of up to 7 MPa [49,50,51,52]. Using intense ultrasound waves one can generate bubble clouds, where individual bubbles interact with each another experiencing translational motion, collision and coalescence. Under certain experimental conditions, these complex bubble interactions may create intriguing structures called streamers (Figure 1e) [34,48], where bubbles nucleate on ‘motes’ that are impurities in the liquid (e.g., solid micro-particles).

Thus, very high pressure levels and shock waves produced as a result of the bubble collapse near the surface of a biological cell are, in general, harmful for the cell. Consequently, this specific regime of bubble oscillations is avoided in biosensing applications that we discuss below. Nevertheless, water jets and streamers (Figure 1c–e) have been safely used to deliver drugs through a blood-brain barrier (BBB) [9,10] (see Figure 1b, a bubble in the middle) that protects brain against circulating toxins and pathogens causing its infections [53]. Many current research efforts are focused on the development of new approaches to modulating and bypassing BBB for therapeutic purposes. Since collapsing gas bubbles have been shown to be safe for controlling BBB, it is plausible that they could also be used to probe mechanical properties of biological cells and tissues. For example, the direction of the water jet produced by a collapsing bubble depends on mechanical properties of the surface near which the bubble oscillates. The bubble develops a jet directed towards a rigid surface but away from a liquid-air interface and it splits near an elastic wall [34,49,50] enabling collection of quantitative information about the elastic properties of the wall. Collapsing bubble properties summarised in Figure 1c–e have also been used in other types of microfluidic biosensors, miniaturised pumps, mixers, filters, transporters and propellers [17,54], which indicates a great application potential of collapsing bubbles in biosensing.

Having noted this, the subsequent discussions in this paper are concerned with predominantly spherical bubble oscillations. They are organised as follows. In Section 2, we outline key mathematical models used to study the dynamics of a single ultrasound-driven spherical bubble in a bulk of liquid. We show that, although the assumed bubble sphericity and fluid incompressibility are an idealisation, models based on it satisfactorily explain experimental observations made in the systems, where many gas bubbles interact with each other in a large cluster and when a single bubble oscillates near an elastic wall or is attached to it. In Section 3, we review methods of determining mechanical properties of cells, bacteria and biological tissues. In particular, oscillating bubbles are considered near a single biological cell. In Section 3.1 and Section 3.2 readers will also find relevant discussions of Brillouin light scattering and scanning acoustic microscopy, respectively. Section 4 introduces the concept of acoustical frequency combs (AFCs) generated using bubble oscillations and shows that AFC-based techniques can extend capabilities of bubble-based sensors. Related approaches are overviewed in Section 3. The applications of gas bubbles in photoacoustic and acousto-optical biosensors are reviewed in Section 5. This is followed by a discussion of applications of artificial intelligence methods in bubble-based biomechanical sensing in Section 6.

Although we aim to make the mainstream presentation self-consistent and accessible to non-experts in acoustics and fluid dynamics, readers wishing to familiarise themselves with a detailed discussion of acoustic waves and their applications in biosensing are recommended to refer to the textbook [55] on fundamentals of acoustics and to the recent review articles on acoustic biosensing [56,57,58,59,60,61].

## 2. Physics of Acoustically Driven Bubble Oscillations

The analysis of physical properties of gas bubbles in liquids has been an active research field since the pioneering work of Lord Rayleigh that explained the cause of damage experienced by propellers of boats and submarines [62]. In that study, the process of cavitation, where gas bubbles are generated in water as a result of changes in the local pressure, was described. The original theory developed by Rayleigh was refined by Plesset [63], Prosperetti [64] and other researchers in the fields of acoustics, fluid dynamics and nonlinear physics [1,2,22,46,65,66,67,68,69,70,71]. The present section summarises the modern theory of oscillating bubbles with a focus on its applications in the areas of biosensing discussed in this current review article.

### 2.1. Acoustically Driven Oscillations of a Single Bubble in Unbounded Liquid

The problem of bubble oscillations in a biological medium is difficult to solve because it involves many physical processes that include acoustic nonlinearities, cavitation processes, compressibility of the hosting liquid, the interaction between individual bubbles in a bubble cluster and that with nearby objects. The latter is of special interest to the field of biological sensing because many biological fluids and tissues may contain naturally trapped bubbles [6,7] and can tolerate the introduction of artificially created bubbles that serve as a contrast agent for ultrasound imaging procedures [12,30,72,73] and are used for drug delivery [9,74]. Subsequently, for the sake of didacticism, we start our discussion with considering a single bubble in unbounded liquid introducing several physical assumption explained next.

**Figure 3:**
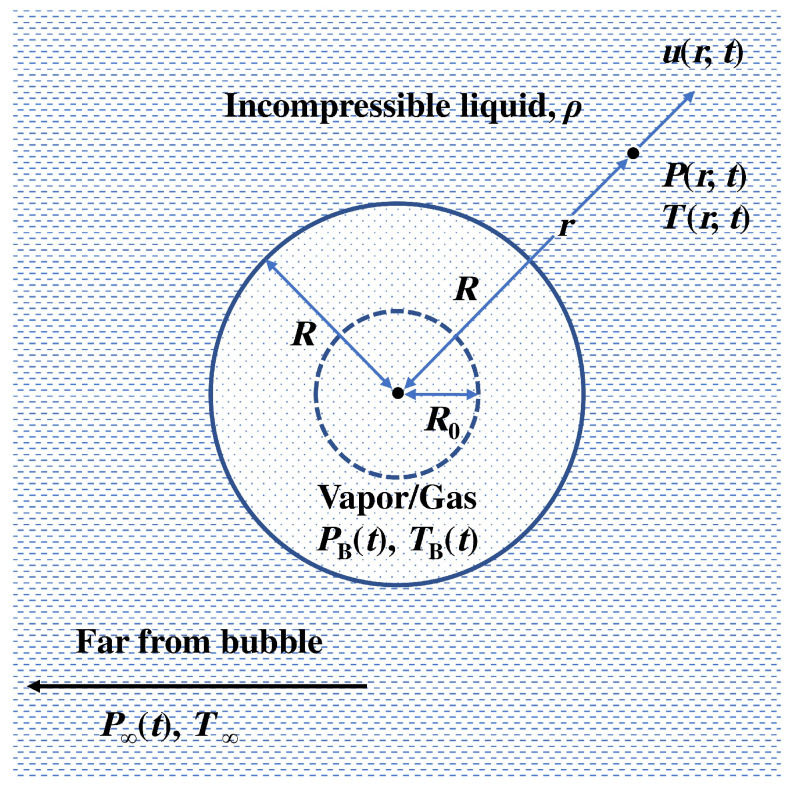
Schematic of a spherical bubble in the bulk of liquid. Parameters used to derive the RP equation are defined in the text.

The fundamental model of oscillations of a spherical bubble driven by an acoustic pressure field in the bulk of liquid is given by the well-known Rayleigh-Plesset (RP) equation. The RP equation for bubble radius R(t) depending on time *t* can be derived under the following assumptions [1]. The bubble exists in an infinite space occupied by a Newtonian fluid (Figure 3), where the temperature and pressure far away from the bubble are $T_{\infty }$ and $P_{\infty }\left(t\right)$, respectively. In the absence of heat sources, $T_{\infty }$ is assumed to be constant. Further, we assume that $P_{\infty }\left(t\right)$ is a known function of time and neglect the compressibility of the liquid. This implies that the liquid density ρ remains constant. The dynamic viscosity μ of a fluid is assumed to be constant as well. The gas contained in a bubble is considered homogeneous. Its temperature $T_{B}\left(t\right)$ and pressure $P_{B}\left(t\right)$ remain uniform, though we note that this condition can be violated if an internal shock or rarefaction wave is created inside a rapidly collapsing or expanding bubble [75].

We denote the distance from the centre of the bubble by *r* and consider the pressure P(r,t), radial outward velocity u(r,t) and temperature T(r,t) of a fluid. Using the conservation of mass we write for an incompressible fluid

(1) $$u\left(r,t\right)=\frac{F(t)}{r^{2}}\;.$$

If there is no mass transport across the bubble surface, the velocity at the interface must satisfy

(2) $$u\left(R,t\right)=\dot{R}=\frac{F(t)}{R^{2}}\;or\;F\left(t\right)=R^{2}\dot{R}\;,$$

where the overdot denotes $\frac{d}{dt}$. If liquid evaporates into a bubble or if its vapour with density $\rho_{V}$ condensates on the bubble wall or diffuses through it, the rate of mass variation inside the bubble of volume *V* is

(3) $${\dot{m}}_{V}=\rho_{V}\dot{V}=\rho_{V}\frac{d(4\pi R^{3}/3)}{dt}=4\pi \rho_{V}R^{2}\dot{R}\;.$$

If *u* is the radial velocity of the liquid at the bubble surface r=R, then the liquid mass flux there is given by

(4) $$\dot{m}=\rho Au=4\pi \rho R^{2}u\;,$$

where *A* is the surface area of the bubble. The conservation of mass then requires that ${\dot{m}}_{V}=\dot{m}$ and $u=\frac{\rho_{V}}{\rho }\dot{R}$. Subsequently,

(5) $$\begin{array}{ccc} u(R,t) & = & \dot{R}-u=\dot{R}-\frac{\rho_{V}}{\rho }\dot{R}=\left(1-\frac{\rho_{V}}{\rho }\right)\dot{R}\;\mathrm{and} \end{array}$$

(6) $$\begin{array}{ccc} F(t) & = & \left(1-\frac{\rho_{V}}{\rho }\right)R^{2}\dot{R}\;. \end{array}$$

Since the liquid density is much larger than that of a vapour, $\frac{\rho_{V}}{\rho }\approx 0$ and $F\left(t\right)\approx R^{2}\dot{R}$.

Subsequently,

(7) $$u\left(r,t\right)=\frac{F(t)}{r^{2}}=\frac{R^{2}}{r^{2}}\dot{R}\;.$$

Navier–Stokes equation describing the radial motion of an incompressible Newtonian fluid is

(8) $$\rho \left(\frac{\partial u}{\partial t}+u\frac{\partial u}{\partial r}\right)=-\frac{\partial P}{\partial r}+\mu \left[\frac{1}{r^{2}}\frac{\partial }{\partial r}\left(r^{2}\frac{\partial u}{\partial r}\right)-2\frac{u}{r^{2}}\right]\;.$$

Then after introducing the kinematic viscosity of the liquid $\nu =\frac{\mu }{\rho }$ we obtain

(9) $$-\frac{1}{\rho }\frac{\partial P}{\partial r}=\frac{\partial u}{\partial t}+u\frac{\partial u}{\partial r}-\nu \left[\frac{1}{r^{2}}\frac{\partial }{\partial r}\left(r^{2}\frac{\partial u}{\partial r}\right)-2\frac{u}{r^{2}}\right]\;.$$

and, using (7),

(10) $$-\frac{1}{\rho }\frac{\partial P}{\partial r}=\frac{1}{r^{2}}\left(2R{\dot{R}}^{2}+R^{2}\ddot{R}\right)-2\frac{R^{4}}{r^{5}}{\dot{R}}^{2}\;.$$

Integrating this equation from the bubble surface r=R to r→∞ produces

(11) $$\begin{array}{ccc} -\frac{1}{\rho }\int_{P(R)}^{P_{\infty }}dP & = & \frac{P\left(R\right)-P_{\infty }}{\rho }\\ & = & \int_{R}^{\infty }\left[\frac{1}{r^{2}}\left(2R{\dot{R}}^{2}+R^{2}\ddot{R}\right)-\frac{2R^{4}}{r^{5}}{\dot{R}}^{2}\right]dr\\ & = & {\left[-\frac{1}{r}\left(2R{\dot{R}}^{2}+R^{2}\ddot{R}\right)+\frac{R^{4}}{2r^{4}}{\dot{R}}^{2}\right]}_{R}^{\infty }=R\ddot{R}+\frac{3}{2}{\dot{R}}^{2}\;. \end{array}$$

**Figure 4:**
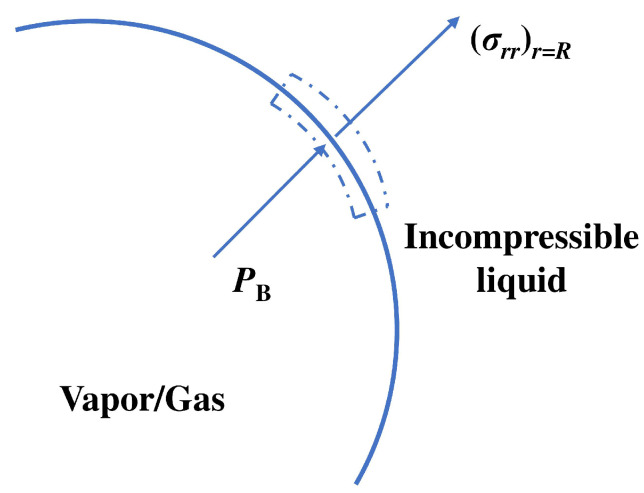
A portion of a spherical bubble surface with physical parameters used to derive the RP equation.

The normal stress $\sigma_{rr}$ in a liquid with constant density and viscosity written in spherical coordinates (see Figure 4) is

(12) $$\sigma_{rr}=-P+2\mu \frac{\partial u}{\partial r}\;.$$

Therefore, the net force per unit area acting on the segment of a bubble surface is

(13) $$\begin{array}{ccc} \sigma_{rr}\left(R\right)+P_{B}-\frac{2\sigma }{R} & = & -P\left(R\right)+{\left.2\mu \frac{\partial u}{\partial r}\right|}_{r=R}+P_{B}-\frac{2\sigma }{R}\\ & = & -P\left(R\right)+2\mu \frac{\partial }{\partial r}{\left(\frac{R^{2}}{r^{2}}\dot{R}\right)}_{r=R}+P_{B}-\frac{2\sigma }{R}\\ & = & -P\left(R\right)-\frac{4\mu }{R}\dot{R}+P_{B}-\frac{2\sigma }{R}\;, \end{array}$$

where σ is the surface tension and $P_{B}$ is the pressure inside a bubble. If there is no mass transfer across the bubble surface, then this force must be zero so that

(14) $$P\left(R\right)=P_{B}-\frac{4\mu }{R}\dot{R}-\frac{2\sigma }{R}\;.$$

Considering the conservation of momentum we obtain

(15) $$\frac{P\left(R\right)-P_{\infty }}{\rho }=\frac{P_{B}-P_{\infty }}{\rho }-\frac{4\mu }{\rho R}\dot{R}-\frac{2\sigma }{\rho R}=R\ddot{R}+\frac{3}{2}{\dot{R}}^{2}\;,$$

and

(16) $$\frac{P_{B}\left(t\right)-P_{\infty }\left(t\right)}{\rho }=R\ddot{R}+\frac{3}{2}{\dot{R}}^{2}+4\frac{\nu }{R}\dot{R}+\frac{2\sigma }{\rho R}\;.$$

If mass heat transfer across the bubble surface are negligible, then

(17) $$P_{B}\left(t\right)=P_{v}+P_{g}\;,$$

where $P_{v}$ and $P_{g}$ are the partial vapour and gas pressures inside the bubble, respectively. To derive the expression for $P_{g}$, it is assumed that the gas behaviour in the bubble is polytropic with exponent κ and no heat is transferred between the gas and the environment. Hence, the mechanical work is performed to change the internal thermal energy of the gas inside the bubble and

(18) $$P_{g}V_{R}^{\kappa }=P_{g0}V_{R0}^{\kappa }=const.\;\mathrm{or}\;P_{g}=P_{g0}{\left(\frac{V_{R0}}{V_{R}}\right)}^{\kappa }\;.$$

Since the shape of the bubble is assumed to remain spherical, the volumes of the bubble corresponding to its equilibrium radius $R_{0}$ and instantaneous radius *R* are $V_{R0}=\frac{4}{3}\pi R_{0}^{3}$ and $V_{R}=\frac{4}{3}\pi R^{3}$, respectively. Therefore,

(19) $$P_{g}=P_{g0}{\left(\frac{R_{0}}{R}\right)}^{3\kappa }\;,$$

where $P_{g0}$ is the gas pressure inside the bubble of the equilibrium radius $R_{0}$. The external pressure $P_{\infty }\left(t\right)$ is the sum of the static pressure $P_{0}$ and a varying pressure, say, p(t)=−αsinωt, where α is the peak amplitude and ω=2πf is the angular frequency of an acoustic pressure wave that forces the bubble.

Assuming further that initially $P_{\infty }\left(0\right)=P_{0}$ and $\dot{R}\left(0\right)=0$ and, consequently,

(20) $$P_{v}-P_{\infty }\left(0\right)+P_{g0}-\frac{2\sigma }{R_{0}}=0\;or\;P_{g0}=P_{0}-P_{v}+\frac{2\sigma }{R_{0}},$$

after substituting the derived expressions for $P_{B}\left(t\right)$ and $P_{\infty }\left(t\right)$ into (16) we obtain the RP equation

(21) $$\begin{array}{ccc} \rho \left[R\ddot{R}+\frac{3}{2}{\dot{R}}^{2}\right] & = & \left(P_{0}-P_{v}+\frac{2\sigma }{R_{0}}\right){\left(\frac{R_{0}}{R}\right)}^{3\kappa }-\frac{2\sigma }{R_{0}}-\frac{4\mu }{R}\dot{R}\\ & & +P_{v}-P_{0}+\alpha \sin\left(\omega t\right)\;. \end{array}$$

This equation does not account for energy losses due to heat conduction into the liquid surrounding a bubble [76] or due to the liquid compressibility that leads to the emission of sound waves by a bubble [76,77].

#### Extensions of Rayleigh-Plesset Equation

Thus far our discussion has not considered damping of bubble oscillations due to acoustic losses. An extended model considering this physical effect was proposed in [78,79], where a term $\frac{\dot{R}}{c}$ (*c* is the speed of sound in a liquid) was introduced to derive the modified RP equation. This term is negligibly small when the motion of the wall of the bubble is much smaller than *c*, but it plays an important role when the wall motion accelerates quickly under strong forcing near the bubble collapse stage. In this case, $\frac{\dot{R}}{c}∼1$, which, mathematically, results in the increase of the order of the so-modified RP equation from second to third. However, this requires an additional initial condition for $\ddot{R}$ that cannot be determined from physical considerations [80].

To resolve this problem, alternative versions of the RP equation were suggested. One of them is Gilmore equation [22,81] that is often used to study the bubble dynamics in the contexts of underwater explosions and geophysical explorations [22]. The key parameter there is not the pressure but the enthalpy. Another popular modification is Keller-Miksis (KM) equation that accounts for the acoustic pressure wave radiation by an oscillating bubble introducing a retarded time $t-\frac{R}{c}$ [82]:

(22) $$\left(1-\frac{\dot{R}}{c}\right)R\ddot{R}+\frac{3}{2}{\dot{R}}^{2}\left(1-\frac{\dot{R}}{3c}\right)=\frac{1}{\rho }\left(1+\frac{\dot{R}}{c}+\frac{R}{c}\frac{d}{dt}\right)\left[P\left(R,\dot{R}\right)-P\left(t\right)\right]$$

with

(23) $$\begin{array}{ccc} P(R,\dot{R}) & = & \left(P_{0}-P_{v}+\frac{2\sigma }{R_{0}}\right){\left(\frac{R_{0}}{R}\right)}^{3\kappa }-\frac{2\sigma }{R_{0}}-\frac{4\mu }{R}\dot{R}\;, \end{array}$$

(24) $$\begin{array}{ccc} P(t) & = & P_{0}-P_{v}+\alpha \sin\left(\omega t\right)\;. \end{array}$$

This equation adequately describes the behaviour of a single bubble in many practical situations, including forcing with high-amplitude acoustic pressure waves and the development of nonlinearities [2,71,83].

### 2.2. Single Bubble Oscillating near a Boundary

As discussed throughout this review article, liquids serve as a good approximation for many biological tissues and cells that have a significant water content. However, elastic properties of biological tissues and cells also strongly affect bubble oscillations, which have been demonstrated, for example, in research work on microbubbles used as contrast agents for ultrasound imaging [30,73,74]. Here, we discuss two models that have been used to study the behaviour of a single bubble near solid and elastic walls. Both models are relevant to the mainstream discussion in this review article because in a real-life biosensing scenario a bubble may interact both with biological tissues, cells and bacteria (Section 3.3.2) and solid objects such as substrates that hold a biological specimen under study.

#### 2.2.1. Bubble Oscillating near a Solid Wall

**Figure 5:**
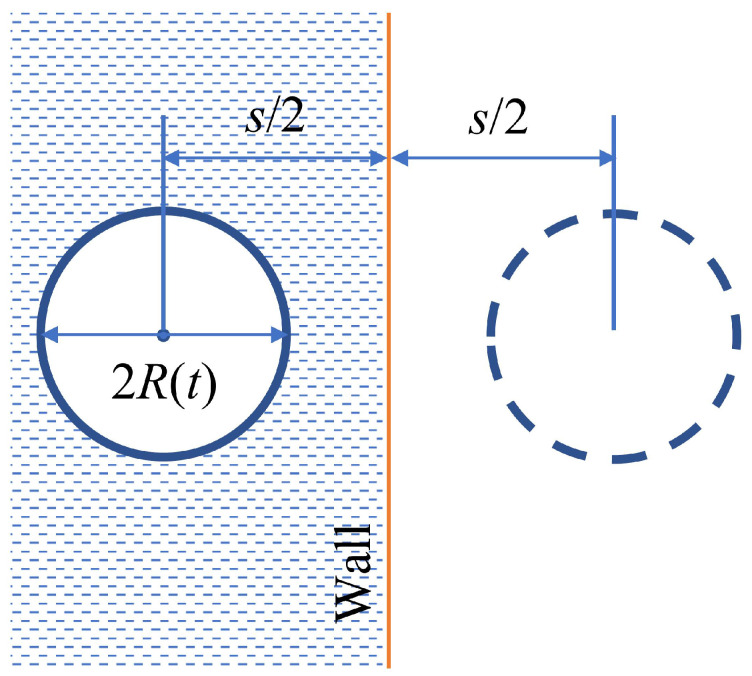
Schematic view of a bubble near a solid wall. The bubble on the right is the ‘mirror’ image of the real bubble.

The nonlinear response of a bubble near a solid wall can be studied using the modified KM equation [65,82,83,84]. Since viscous forces acting in the fluid near the wall are neglected, one can use the potential flow theory to replace the wall with an identical ‘mirror’ bubble image that is located symmetrically with respect to the rigid wall and oscillates with the same frequency, amplitude, and phase as the original bubble [71,83]. Even though in reality Bjerknes forces could cause the distance $\frac{s}{2}$ between the bubble centre and the wall vary [67,85,86,87] (see also Section 2.3), for simplicity it is often assumed that it remains constant (Figure 5). The corresponding equation is know as Keller-Miksis-Parlitz equation [65,83].

It reads

(25) $$\left(1-\frac{\dot{R}}{c}\right)R\ddot{R}+\frac{{\dot{R}}^{2}}{2}\left(3-\frac{\dot{R}}{c}\right)=\frac{1}{\rho }\left[1+\frac{\dot{R}}{c}+\frac{R}{c}\frac{d}{dt}\right]\left[P\left(R,\dot{R}\right)-P\left(t\right)\right]-\frac{1}{s}\left(R^{2}\ddot{R}+2R{\dot{R}}^{2}\right)\;.$$

#### 2.2.2. Bubble Oscillating near an Elastic Wall

**Figure 6:**
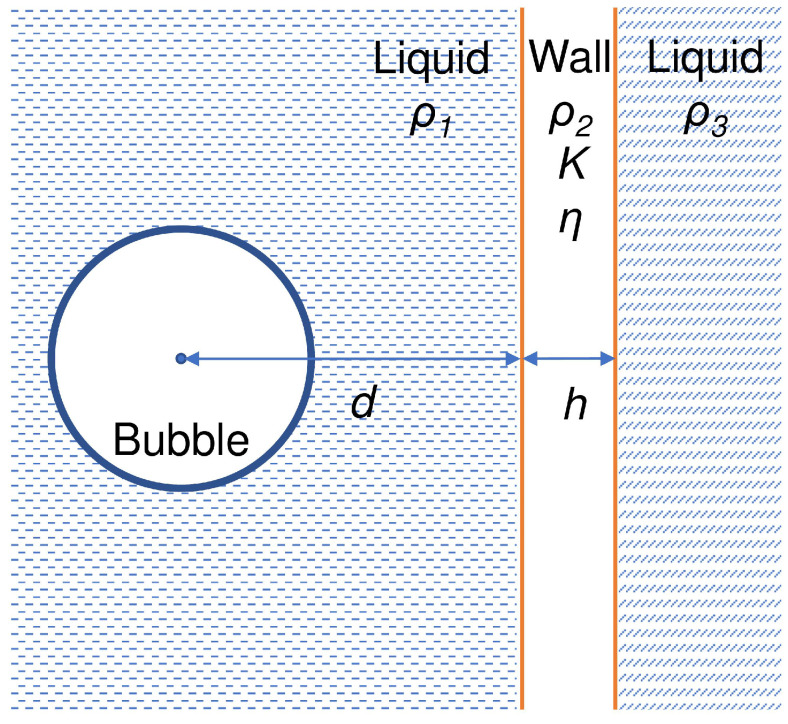
Schematic of a spherical bubble near an elastic wall.

Even though the model given by Equation (25) assumes that the wall is rigid, it accurately predicts the dynamics of an oscillating bubble when it approaches a blood vessel wall. However, when the distance to the wall becomes of the order of the bubble radius, the wall elasticity can have a noticeable effect and the wall can deform. Thus, another model accounting for the elasticity and finite thickness of the wall has been developed [30]:

(26) $$\begin{array}{ccc} & & R\ddot{R}\left[1-\left(\frac{\rho_{1}-\beta }{\rho_{1}+\beta }\right)\frac{R}{2d}-\left(\frac{\beta -\rho_{3}}{\beta +\rho_{3}}\right)\frac{R}{2(d+h)}+\frac{\left(\rho_{1}-\beta \right)}{\left(\rho_{1}+\beta \right)}\frac{\left(\beta -\rho_{3}\right)}{\left(\beta +\rho_{3}\right)}\frac{R}{2h}-\frac{\dot{R}}{c}\right]\\ & & \;+\frac{3}{2}{\dot{R}}^{2}\left[1-\left(\frac{\rho_{1}-\beta }{\rho_{1}+\beta }\right)\frac{2R}{3d}-\left(\frac{\beta -\rho_{3}}{\beta +\rho_{3}}\right)\frac{2R}{3(d+h)}+\frac{\left(\rho_{1}-\beta \right)}{\left(\rho_{1}+\beta \right)}\frac{\left(\beta -\rho_{3}\right)}{\left(\beta +\rho_{3}\right)}\frac{2R}{3h}-\frac{\dot{R}}{3c}\right]\\ & & =\frac{1}{\rho_{1}}\left(1+\frac{\dot{R}}{c}+\frac{R}{c}\frac{d}{dt}\right)\left[\left(P_{0}+\frac{2\sigma }{R_{0}}\right){\left(\frac{R_{0}^{3}-a^{3}}{R^{3}-a^{3}}\right)}^{\kappa }\right.\\ & & \;\left.-\frac{2\sigma }{R}-\frac{4\mu }{R}\dot{R}-P_{0}-\alpha \sin\left(\omega t\right)\right]\;, \end{array}$$

where *a* is the radius of the bubble’s van der Waals hard core, *c* is the speed of sound in the liquid surrounding bubble, $\beta =\rho_{2}\frac{3K-2\eta }{3K+4\eta }$, *K* and η are the bulk and shear moduli of the wall material, respectively, *h* is the thickness of the wall, *d* is the distance between the centre of the bubble and the wall and $\rho_{1}$, $\rho_{2}$ and $\rho_{3}$ are the densities of the liquid surrounding the bubble, of the wall and of the liquid behind the wall, respectively (see Figure 6).

It has been demonstrated using Equation (26) that the natural frequency of a bubble can decrease or increase depending on the material properties of an elastic wall, which is relevant to the discussion in Section 3.3.2. The presence of the wall also affects acoustic power scattered by the bubble [30]. Therefore, mechanical properties of biological tissues and individual cells can be determined by analysing responses of bubbles oscillating in their proximity.

### 2.3. Interaction of Oscillating Bubbles in a Bubble Cluster

Up to this point, we have considered a stationary single bubble oscillating either in the bulk of water or near a wall. However, in many practical situations one encounters multiple bubbles of generally different sizes grouped into a cluster. When such a cluster is insonated with an ultrasound pressure wave, bubbles in the cluster both oscillate and interact with each other in a very complex way leading to their translation relative to their initial positions. The physics of bubbles cluster behaviour has been a subject of intensive research [67,88,89,90,91,92,93,94,95,96,97,98]. Most of such studies are based on the accepted models of spherical bubble oscillations that have been reviewed in the preceding sections. In addition, to account for translational motion, the models developed in the cited works considered the impact of Bjerknes forces [99] that act on oscillating bubbles. The primary Bjerknes force $F_{pB}$ is caused by the imposed acoustic pressure field [99,100] while the secondary Bjerknes force $F_{sB}$ arises between two or more interacting bubbles [46]. For example, the secondary Bjerknes force between two bubbles is repulsive when the driving frequency lies between bubbles’ natural frequencies; otherwise, it is attractive [46,101,102].

To investigate the interaction between multiple bubbles in a cluster, Equation (21) can be extended using a term representing the pressure that arises due to scattering of the incident ultrasound pressure wave by the neighbouring bubbles [67]. This extended model can be further modified to account for bubble translation see, for example, [103]. The resulting system of differential equations reads

(27) $$\begin{array}{ccc} & & R_{n}{\ddot{R}}_{n}+\frac{3}{2}{\dot{R}}_{n}^{2}-\frac{P_{n}}{\rho }=\frac{{\dot{p}}_{n}^{2}}{4}\\ & & \;-\sum_{\begin{array}{c} l=1\\ l\neq n \end{array}}^{N}\left\{\frac{R_{l}^{2}{\ddot{R}}_{l}+2R_{l}{\dot{R}}_{l}^{2}}{d_{nl}}+\frac{R_{l}^{2}}{2d_{nl}^{3}}\left(p_{n}-p_{l}\right)\cdot \left(R_{l}{\ddot{p}}_{l}+{\dot{R}}_{l}{\dot{p}}_{n}+5{\dot{R}}_{l}{\dot{p}}_{l}\right)\right.\\ & & \;\;\left.-\frac{R_{l}^{3}}{4d_{nl}^{3}}\left[{\dot{p}}_{l}\cdot \left({\dot{p}}_{n}+2{\dot{p}}_{l}\right)+\frac{3}{d_{nl}^{2}}\left[{\dot{p}}_{l}\cdot \left(p_{l}-p_{n}\right)\right]\left[\left(p_{n}-p_{l}\right)\cdot \left({\dot{p}}_{n}+2{\dot{p}}_{l}\right)\right]\right]\right\}\;, \end{array}$$

(28) $$\begin{array}{ccc} & & \frac{1}{3}R_{n}{\ddot{p}}_{n}+{\dot{R}}_{n}{\dot{p}}_{n}=\frac{F_{n}}{2\pi \rho R_{n}^{2}}+\sum_{\begin{array}{c} l=1\\ l\neq n \end{array}}^{N}\left\{\frac{\left(p_{n}-p_{l}\right)B_{1}}{d_{nl}^{3}}-\frac{R_{l}^{2}}{2d_{nl}^{3}}\left(R_{n}R_{l}{\ddot{p}}_{l}+B_{2}{\dot{p}}_{l}\right)\right.\\ & & \;\left.+\frac{3R_{l}^{2}}{2d_{nl}^{5}}\left(p_{n}-p_{l}\right)\left[\left(p_{n}-p_{l}\right)\cdot \left(R_{n}R_{l}{\ddot{p}}_{l}+B_{2}{\dot{p}}_{l}\right)\right]\right\}\;, \end{array}$$

where $p_{l}$ is the position vector of the *l*th bubble, $d_{nl}$ is the distance between bubbles *l* and *n*, $B_{1}=R_{n}R_{l}^{2}{\ddot{R}}_{l}+2R_{n}R_{l}{\dot{R}}_{l}^{2}+{\dot{R}}_{n}{\dot{R}}_{l}R_{l}^{2}$ and $B_{2}={\dot{R}}_{n}R_{l}+5R_{n}{\dot{R}}_{l}$. Equation (27) describes the radial oscillations of the *n*th bubble in the cluster and Equation (28) governs its translational motion. The pressure $P_{n}$ is defined as

(29) $$P_{n}\left(R,\dot{R}\right)=\left(P_{0}-P_{v}+\frac{2\sigma }{R_{n0}}\right){\left(\frac{R_{n0}}{R_{n}}\right)}^{3\kappa }-\frac{4\mu }{R_{n}}{\dot{R}}_{n}-\frac{2\sigma }{R_{n}}-P_{0}-P_{v}-P_{ex}\left(p_{n}\right)\;,$$

where $P_{ex}\left(p_{n}\right)$ is the pressure of the driving ultrasound wave in the centre of the *n*th bubble. The external forces $F_{n}$ are equal to the primary Bjerknes force

(30) $$F_{nB}=-\frac{4\pi }{3}R_{n}^{3}\nabla P_{ex}\left(p_{n}\right)$$

and the force exerted on the bubble by the surrounding fluid (the Levich viscous drag [104]) is given by

(31) $$F_{nL}=-12\pi \mu R_{n}\left({\dot{p}}_{n}-\sum_{\begin{array}{c} l=1\\ l\neq n \end{array}}^{N}v_{ln}\right)\;,$$

where the liquid velocity generated by the *l*th bubble in the centre of the *n*th bubble is

(32) $$v_{nl}=\frac{R_{l}^{2}{\dot{R}}_{l}\left(p_{n}-p_{l}\right)}{d_{nl}^{3}}+\frac{R_{l}^{3}}{2d_{nl}^{3}}\left\{\frac{3(p_{n}-p_{l})}{d_{nl}^{2}}\left[{\dot{p}}_{l}\cdot \left(p_{n}-p_{l}\right)\right]-{\dot{p}}_{l}\right\}\;.$$

The liquid velocity generated by the driving pressure field in the centre of the *n*th bubble is neglected in Equation (31) since it is small for low peak pressures. This equation was used to produce results discussed in Section 4.2.

## 3. Determining Mechanical Properties of Cells, Bacteria and Biological Yissues

Mechanical properties of biological cells play an important role in the regulation of cell physiology by enabling various kinds of spontaneous motion that a cell can undergo: swimming, crawling, gliding or swarming. They also influence the processes of cell division and adhesion [105,106,107,108,109,110]. In particular, cellular viscoelasticity has attracted close attention of researchers since it changes dramatically when a cell becomes unhealthy [111,112,113]. For example, a direct connection between single-cell biomechanics and cancer has been established [108,114,115,116,117]. Those findings have revealed the potential of using the viscoelastic properties of cells as markers for extracellular characteristics of a tumour [112]—the presence of tumours could be reliably detected by comparing mechanical properties of healthy and diseased cells.

Furthermore, theoretical studies have demonstrated that viruses [118,119,120,121], bacteria [33,122] as well as larger and more complex living organisms [123,124] can behave as mechanical systems that resonate at certain vibrational frequencies. For example, it was calculated that the lowest frequency of a spherical virus with a 50 nm radius is of the order of a few GHz [118,125]. Cells of green algae resonate at about 1 MHz [126]. There have also been reports of resonance-like phenomena observed in HeLa cells at the frequency of 750 kHz [127] and of a role bubbles play in acoustically-induced cell lysis (breaking off a cellular membrane) [128].

In the case of more complex living organisms, it has been shown that an earthworm can resonate at mechanical vibration frequencies of about 100 Hz. Given that vibrations can be induced in a very broad frequency range using suitable equipment (e.g. mechanical oscillators, ultrasonic piezoelectric transducers and pulsed lasers [121,129]), it is plausible that pathogens such as bacteria and viruses can be deactivated by breaking their body structures using high-amplitude resonances. Similar procedures employing a less intense mechanical stimulation could be employed to non-destructively measure elastic properties of individual cells as well as of larger living organisms such as worms. In the latter case, such measurements would enable assessing elastic properties of worm’s internal organs and tissues, the knowledge of which is important for applications in experimental biology [123] and for the development of novel methods that exploit earthworms to increase soil fertility [130].

However, despite the maturity of existing techniques [110,117,129,131,132,133,134,135,136,137], most of them measure only mechanical properties of cell membranes or their combinations with cytoskeleton. As a result, the information about mechanical properties of the interior of an individual cell may be difficult to gain. In turn, this complicates the detection of tumours and other abnormalities. A similar problem has been identified in the field of worm biology: the presently used experimental techniques mostly enable measuring the stiffness of worm cuticle [123,124] while the overall elastic properties of a worm also depend on those of internal organs and tissues [124].

In this section, we review measurement techniques that can provide information about mechanical properties of the interior of individual cells.

### 3.1. Brillouin Light Scattering Spectroscopy

Brillouin light scattering (BLS) is a physical effect named after Léon Brillouin, where the interaction of light with material waves in a medium arises due to by the dependence of the optical refraction index (the ratio of the speed of light in a vacuum to the phase velocity of light in the medium) on the properties of the medium [125,138]. The refraction index of a transparent material changes when it is mechanically deformed and a small fraction of light that is transmitted through the material or reflected from it changes its momentum (its energy is changed and thus the frequency is shifted by the amount called Brillouin shift [125,138]). This process is similar to a light diffraction by grating, the components of which vibrate with a frequency that is much smaller than that of a light wave. In biological media and liquids, BLS can be observed as a result of light interaction with acoustic (phononic) modes existing in the GHz acoustic spectral range, thus providing a nondestructive contactless micro-scale probe of the mechanics of biological cells [125,129,134,139,140,141]. Brillouin light scattering spectroscopy is one of the techniques that enable investigating mechanical properties of an individual cell’s interior [23,129,139,140].

**Figure 7:**
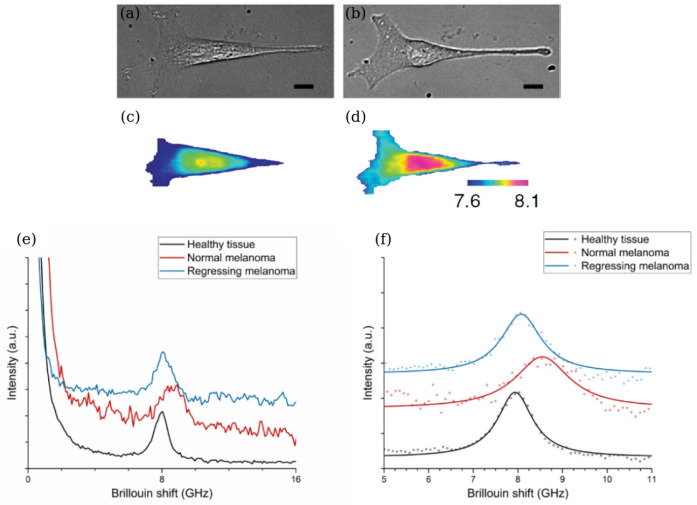
(**a**,**b**) Phase-contrast microscopy and (**c**,**d**) co-registered BLS spectrospcopy-based images of a mouse fibroblast cell before (**a**,**c**) and after (**b**,**d**) a hyperosmotic shock. The scale bars of the phase contrast images are 10 μm. The false colour map of the BLS-based images encode the measured Brillouin frequency shifts in the range from 7.6 to 8.1 GHz. Reproduced from [139] under the terms of a Creative Commons Attribution (CC-BY) License. (**e**) Representative anti-Stokes Brillouin peaks of the BLS spectra obtained for a healthy tissue (black curve), normal non-regressing melanoma (red curve) and regressing melanoma (blue curve). (**f**) Close-up view of the spectra shown in panel (**e**): dots correspond to the raw experimental data and lines show the respective Lorentzian function fit of the raw data. Reproduced from [23]. Copyright 2019 Optical Society of America under the terms of the OSA Open Access Publishing Agreement.

Figure 7a–d compares the phase contrast and BLS spectroscopy-based images of a mouse fibroblast cell before and after a hyperosmotic shock that was induced by adding 50 mM of sucrose [139]. The images reflect the frequency shifts between the incident and reflected lights caused by the BLS effect and encoding them using a false colour map. The so-obtained images reveal a readily detectable increase in the Brillouin frequency shift throughout the body of a cell that can be related to the difference between the longitudinal elastic modulus of the cell organelles and that of water accounting for more than 70% of the total cell mass and producing a Brillouin frequency shift peak at approximately 7.5 GHz. This corresponds to a bulk modulus of approximately 2.2 GPa [139].

The ability of BLS spectroscopy to determine mechanical properties of the interior of a biological cell has been used to distinguish between healthy and cancerous regions of melanoma excised from a Sinclair miniature swine [23]. Figure 7e shows data obtained in a BLS measurement of non-regressing and regressing melanomas and of a healthy tissue region. One can see that the values of the signal-to-noise ratio (SNR) for the three BLS spectra are different despite the same data acquisition parameters used in the measurements. In particular, a healthy tissue possesses the largest value of SNR while a signal from non-regressing melanoma has the smallest SNR. This difference was explained by an increased absorption of the incident light by melanin—a natural skin pigment—that is encountered in high concentrations in tissues affected by the non-regressing melanoma. The raw Brillouin peaks were fit with a Lorentzian function to obtain their central frequencies and to resolve the peak linewidths (Figure 7f). One can see that changes in the Brillouin frequency shift are accompanied by variations of the peak linewidth, which implies that the latter can also be used to analyse data. It is also seen that the values of Brillouin frequency shifts in Figure 7e are close to those in Figure 7a–d that were obtained using different biological samples.

In fact, many biological cells have a high water content and similar mechanical properties, which implies that the currently available experimental approaches require improvement to be resole very small property differences between them. Being a non-contact measurement technique, BLS spectroscopy enables investigating such properties with a high spatial resolution. However, several technological difficulties have to be resolved to unlock the full potential of this technique and to render it to commercial applications. Firstly, the equipment used in current BLS setups is complex, fragile and expensive. Therefore, it cannot be delivered to end-users in clinical settings. In fact, while a user-friendly data acquisition and handling interface can be designed for a BLS setup [140], the problem of mechanical robustness of any optical equipment, which employs a Fabry–Perot interferometer and delicate moving components, still awaits its resolution. In addition, protocols for sample preparation and measurement should be be standardised and consolidated across a range of instrument designs [140]. In particular, reliable statistical methods and software based on them need to be developed to differentiate BLS images of healthy and abnormal tissues, which, in turn, requires larger-scale clinical trials.

### 3.2. Scanning Acoustic Microscopy

Scanning acoustic microscopy (SAM) is another technique that enables studying mechanical properties of an individual cell interior with a spatial resolution of a few micrometres [110,142] similar to that of the BLS spectroscopy (Section 3.1). Since its introduction in the early 1970s, SAM has been used to investigate elastic properties of biological cells. One of its advantages is its safety. Unlike BLS spectroscopy, it does not cause potential photodamage by a prolonged illumination of a cell by light [40]. Moreover, using SAM does not require any staining of a cell that is needed in fluorescence microscopy to improve cell visualisation [40].

**Figure 8:**
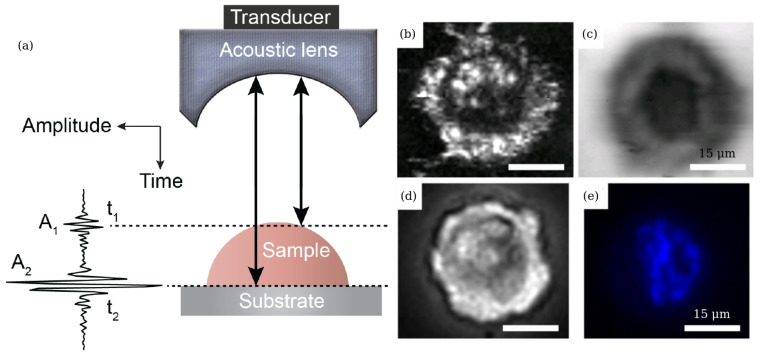
(**a**) Illustration of the operating principles of SAM. The acoustic echo signal with amplitude $A_{1}$ originating from the surface of the sample arrives at time $t_{1}$. The echo signal with amplitude $A_{2}$ is reflected from the interface between the sample and its substrate and is received at time $t_{2}$. From the arrival time of each maximum and their respective amplitudes, the elastic and mechanical parameters of the sample can be obtained. (**b**) Image of MCF-7 breast cancer cells obtained using SAM at 1 GHz acoustic frequency. (**c**) Acoustic wave attenuation distribution across a cell. (**d**) Optical micrograph of a cell. (**e**) Fluorescence-based micrograph of the same MCF-7 cell, where the stained cell nucleus overlaps with the darker area in the image in panel (**c**). The scale bar in all panels corresponds to 15 μm. Reproduced from [142] under the terms of the Creative Commons Attribution 4.0 International Public License (CC-BY 4.0).

While there exist various variants of SAM [110,142], in general its setup uses an acoustic pulse that is directed to and focused on a sample by a piezoelectric transducer combined with an acoustic lens (Figure 8a). The same transducer-lens combination transmits acoustic pulses and receives echoes reflected from a sample and a substrate holding it [110,142]. Transition between the pulse emission and echo reception modes is achieved using a fast electronic switch. A liquid that serves as a buffer medium is used to acoustically couple the lens with the sample.

In particular, SAM was used to produce images of chicken heart fibroblasts that were taken at the frequency of 1.5 GHz. They revealed several prominent features such as dark streaks along the cell boundary [143]. A SAM-based noninvasive procedure for ultrasound imaging was used to investigate the structure of fish embryos and to measure the speed of sound in their tissues [144]. A number of important physical parameters, including cell thickness, sound speed, acoustic impedance, density, attenuation and bulk modulus of MCF-7 breast cancer cells were reported in [142,145]. Figure 8 shows their acoustic images in interphase and metaphase alongside the corresponding optical and fluorescence images of the cell. They reveal an increase in thickness during cells transition between the two phases. Study [145] also demonstrated that the transition of cells from early to late apoptosis was accompanied by a decrease in the cell thickness, which is consistent with the previously reported results for cancer cells undergoing division.

Thus, SAM enables one to image the interior of an individual cell using technically simple equipment compared with BLS spectroscopy reviewed in Section 3.1. However, SAM also has drawbacks that originate from fundamental physical limitations such as large acoustic attenuation at GHz-range acoustic frequencies [125] and the need for specialised piezo-electric transducers and driving electronics capable of operating in that range. Any further development of SAM towards commercial applications requires large-scale clinical trials, which is also the case for BLS spectroscopy.

To resolve these problems, it has been suggested that the capabilities of BLS and SAM could be combined into a single device that employs a thin-film-based opto-acoustic transducer for generating and detecting GHz-range acoustic waves in the sample [146,147,148,149]. Unlike in a standard BLS spectroscopy setup, a mechanical method of acoustic wave excitation eliminates the need for illuminating the studied cell using intense laser pulses that can cause cell damage. At the same time, compared with the standard SAM technique, optical detection enhances the signal-to-noise ratio (SNR) and improves image quality, which was demonstrated using both phantom cells and fixed fibroblast cells in [146,147].

### 3.3. Deformation of Cells and Bacteria

Despite all improvements of BLS spectroscopy and SAM techniques that have been achieved thus far, situations still exist in which mechanical properties of cells and similar biological objects cannot be determined using them due to fundamental physical limitations. In this section, we demonstrate that some of such limitations can be overcome using unique acoustic properties of gas bubbles in liquids.

#### 3.3.1. Mechanical Resonance Properties of Cells And Bacteria

To start with, we note a well-established fact that many biological cells and bacteria are mechanical oscillation systems. This fact was first pointed out in [150], where resonance frequencies and resonance quality factors of red blood cells were calculated using an idealised mathematical model representing a cell as a spherical, isotropic elastic shell filled with and surrounded by viscous fluids. However, several algebraic errors made in [150] limit potential applicability of the results presented there (see [122] for a relevant discussion). On the other hand, this and followup studies by the same author [151] provided useful insight into physical phenomena underpinning mechanical oscillations of biological cells.

A more rigorous theory was developed in [152], where the influence of elasticity of cellular materials on natural oscillations of biological cells was taken into account. In particular, it was demonstrated that oscillations with a high quality factor were possible in biological cells with sufficiently rigid walls, which is the case for some plant cells and bacteria. Those findings are in good agreement with relevant experimental data obtained for algae hydrodiction cells at the frequency of about 1 MHz [126].

In a more recent theoretical work [122], spectra of natural oscillations of different types of bacteria were studied using the model proposed in [152], where modern data for mechanical properties of bacteria were used. In particular, a dispersion relation for mechanical oscillations of a single bacterium was developed based on a core-shell model. It takes into account elasticity of the bacterium shell and viscoelastic properties of liquids inside and outside the bacterium (a similar core-shell model was previously shown to adequately describe the attenuation of acoustic pressure waves in red blood cell suspensions [153]). Using this model, it was demonstrated that the characteristic natural motion of an individual biological cell is determined by the elastic properties of its shell and viscoelasticity of the surrounding liquid.

**Figure 9:**
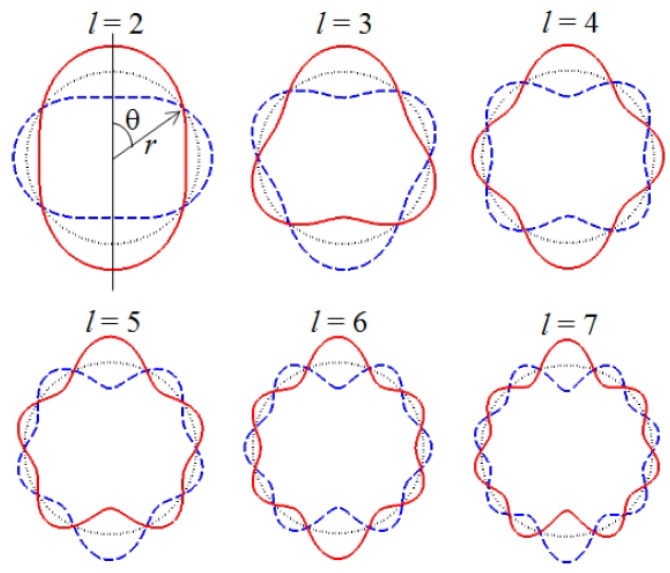
Cross-sections of theoretical 3D shapes assumed by a liquid drop at $T_{l}=0$ (dashed curves), $T_{l}=1$ (solid curves) and $T_{l}=1/2$ (dotted curve), where $T_{l}=\omega_{l}t$ is given in the units of π radians and $T_{l}=1$ corresponds to a half of a drop oscillation period corresponding to mode number *l*.

Before we continue this discussion, we note that in many models of oscillating cells and bacteria their shape is assumed to be spherical and, as the first approximation, they are analysed using the theory developed to explain natural oscillations of liquid drops [154]. Thus, we consider capillary oscillations of a single, initially spherical liquid drop and analyse the natural angular frequencies $\omega_{l}$ and peak oscillation amplitude $A_{l}$, where *l* is the ordering number of the oscillation mode. The frequency $\omega_{l}$ of infinitesimal oscillations of an inviscid drop is given by [155]

(33) $$\begin{array}{c} \omega_{l}^{2}=l\left(l-1\right)\left(l+2\right)\frac{\sigma }{\rho R^{3}}\;, \end{array}$$

where σ and ρ are the surface tension and the density of a liquid, respectively. The modes l=0 and l=1 have zero frequency and correspond to the conservation of volume and the translational invariance, respectively. The lowest (fundamental) oscillating mode excited in experimental conditions is l=2. The three-dimensional shapes of the oscillation modes are given by the expression [156]:

(34) $$r=R\left[1+\frac{A_{l}}{R}\cos\left(\omega_{l}t\right)P_{l}\left(\cos\theta \right)\right]\;,$$

where the coordinate origin is at the centre of the drop (see Figure 9), *t* is time, 0≤θ<2π,

$$P_{l}\left(x\right)=\sum_{m=0}^{M}{\left(-1\right)}^{m}\frac{(2l-2m)!}{2^{l}m!\left(l-m\right)!\left(l-2m\right)!}x^{l-2m}$$

is Legendre polynomial and $M=\frac{l}{2}$ or $M=\frac{l-1}{2}$, whichever is an integer. The viscosity effects were taken into account in [157,158].

Various shapes assumed by an initially spherical drop for the modes l=2,…,7 with the oscillation amplitudes $A_{l}/R=0.3$ [159] are shown in Figure 9. Observe that for the fundamental mode l=2 the shape of the drop changes from an oblate to prolate spheroid (in [122] calculations were carried out for the mode l=2 since it appears to be the most important in the drop breakup [160]). For l=3, the shape of the oscillating drop changes from an inverted pyramid to a pyramid. For higher modes the drop assumes even more complex shapes.

Thus, since the typical BLS shift is of order of several GHz (Section 3.1), using BLS for probing mechanical properties of bacteria and biological cells with natural frequencies in the MHz frequency range is impossible. SAM and similar experimental techniques cannot be applied for this purpose either because mechanical resonances of cells and bacteria are difficult to excite by an acoustic pressure wave. Indeed, based on a model that considers a cell as a liquid drop [154] it was demonstrated that the acoustic scattering cross section of a cell at the frequency of its fundamental shape resonance (l=2 in Figure 9) is very small. For example, the wavelength of the acoustic wave in water at the resonance frequency for a 30 μm-radius spherical bacterium is $\lambda_{a}=1.67$ mm [122], i.e., approximately 55 times larger than the radius *a* of the bacterium. Since the acoustic cross-section of a small particle-like bacterium is proportional to the fourth power of the ratio ${\left(\frac{a}{\lambda_{a}}\right)}^{4}$, the effect of a plain acoustic pressure wave on the bacterium at the frequency of its fundamental shape resonance is negligible (for a relevant discussion of the result of a rigorous numerical modelling see, e.g. [161]). While it has been suggested that a modulated plane acoustic pressure wave, where the wavelength of the carrier wave is close to the radius of the bacterium, might have a higher impact on it [160], this approach has not yet been experimentally verified.

#### 3.3.2. Deformation of Cells and Bacteria by Bubbles

To discuss a potential resolution of the problem with a weak interaction between plane acoustic pressure waves and an individual biological cell or bacterium, we first refer to the already discussed analogy between light and acoustic waves [162]. It is well-known in the field of optics that any standard optical microscope has an ultimate limit in spatial resolution imposed by diffraction of visible light. This restricts the ability of the microscope to distinguish between two objects separated by a distance smaller than approximately half the wavelength of light used to illuminate a sample [163]. This problem has been successfully tackled using nano-emitters such as quantum dots and optical nanoantennas [36,161]. The latter are nano-scale analogues of conventional radio-frequency antennas in terms of their ability to emit and receive electromagnetic waves. A key building block of a typical optical nanoantenna is a metal nanoparticle with a characteristic size that is much smaller than the wavelength of the incident light. Nevertheless, despite its small size such a nanoantenna can efficiently localise the energy of a plane optical wave in a nanoscale volume called ‘hotspot’ due to the property of metal nanoparticles to support localised surface plasmon modes [36,161,164]. Effectively, a nanoantenna acts as a point-like source of radiation that first receives light from a far-field zone and then re-emits it into the near-field zone enabling one to overcome the diffraction limit of light.

**Figure 10:**
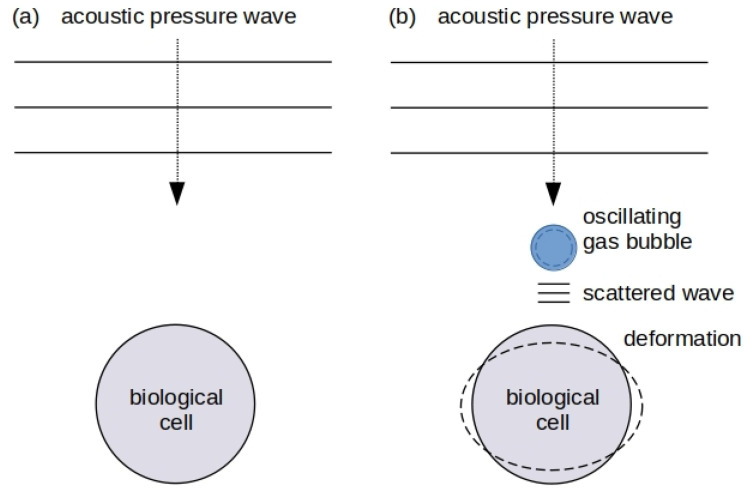
Illustration of the interaction of an oscillating bubble with a biological cell in the ultrasound acoustic pressure field. (**a**) Since a cell typically has a very small acoustic cross-section, the effect of a plain acoustic pressure wave on it at the frequency of its fundamental shape resonance is negligibly small. (**b**) Using a microbubble, one can efficiently excite the shape oscillations of the cell since the bubble acts as a point-like source of acoustic pressure waves. As a result, the cell can be deformed and its mechanical properties can be measured in a non-contact manner.

While in the fields of acoustics and fluid dynamics there are no direct analogues of plasmonic optical modes despite an active search for them [165], unique properties of bubbles oscillating in liquids provide an opportunity to create point-like emitters of acoustic waves that can be used to excite mechanical resonances of biological cells with high efficiency [166]. This conclusion follows from pioneering works by Ackerman [151]. In such an excitation scheme (Figure 10), shape oscillations of a biological cell are driven by an external force that acts uniformly at the scale comparable with the size of a cell and that has a frequency close to the cell resonance [151]. An acoustically driven microbubble oscillating near a cell can serve as a source of such a force [36].

These ideas were further developed in the theoretical work [166], where it was assumed that a bubble oscillates in a stable cavitation regime illustrated in Figure 1a. The suggestion to employ bubbles to deform biological cells and tissues and to probe their mechanical properties also found independent experimental confirmations in [128,167]. In particular, simulations conducted in [166] showed that while the deformation of relatively large bacteria is not sufficient to mechanically break a cell wall, it changes the processes of molecular transport across the cell wall (for a relevant discussion see, e.g., [32]). It was also shown that the acoustic pressure effect of an oscillating bubble can decrease the ability of bacteria to reproduce and infect.

Of course, an increase in the amplitude of the ultrasound wave that drives bubble oscillations may result in well-known effects such as enhanced metabolic productivity of microbial cells in bioreactors [168], sonochemical reactions [19,169] and membrane permeation (sonoporation) [170,171,172]. Under specific conditions, cavitating bubbles may cause a reversible rupture of cell membranes facilitating molecule entry into cells and enhancing the drug delivery [9,128] or the transfer of genetic material into living animal and plant cells [173].

**Figure 11:**
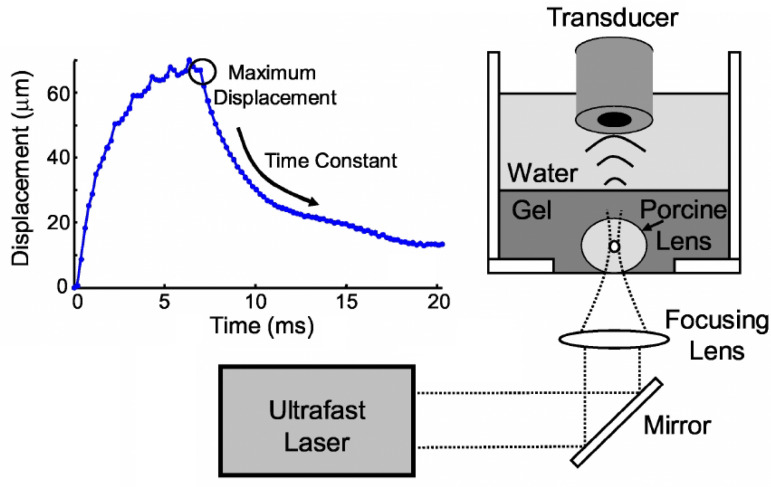
(**Right**) Sketch of an experimental setup for oscillating bubble-based measurements with in vitro porcine eye lenses. Microbubbles are generated across the equatorial plane of a lens using laser-induced optical breakdown. Piezoelectric transducers irradiate bubbles with a MHz-range ultrasound and receive pressure waves scattered by them thereby allowing one to measure both the displacement of bubbles with respect to their initial positions and their temporal responses. (**Left**) Typical experimental bubble displacement curve. Along with the exponential time constant of the bubble temporal responses, knowledge of the maximum displacement enables determining mechanical properties of the lens tissues. Reproduced from [167]. Copyright 2007, with permission from Elsevier.

Operation in a regime, where a bubble undergoes stable small-amplitude oscillations appears to be especially suitable for sensing applications, where a biological cell does not need to be ruptured. Although such sensors have not been purposefully developed yet, there exists experimental evidence that speaks strongly in their favour. For example, microbubbles were generated across the equatorial plane of explanted porcine eye lenses using laser-induced optical breakdown that occurs when femtosecond pulsed laser light interacts with a biological tissue [167,174]. A MHz ultrasonic transducer (Figure 11, right) was used in a cross-correlation method to measure bubble displacements and determine exponential time constants of temporal bubble responses. The maximum bubble displacement (Figure 11, left) is inversely proportional to Young’s modulus of the tissue while the time constants are indicative of the tissue’s viscoelastic properties. It was found that the bubble displacement decreases both near the porcine lens centre and with lens age. This suggests that the nucleus is stiffer than cortex and that porcine lenses become stiffer with age. Therefore, the resulting bubble-based acoustic radiation sensor may be well-suited as a potential in vivo technique to create spatial maps of elastic properties of the lens and guide therapeutic procedures aimed at restoring sight [167]. We will continue this discussion in Section 4.3.

## 4. Acoustic Frequency Combs

Optical frequency combs (OFCs) are optical spectra that contain equidistant coherent frequency peaks [175]. OFC-based technologies have enabled researchers and engineers to measure frequencies of complex signals with high precision, thereby revolutionising the areas of sensing, metrology and communications and benefiting fundamental science [175]. However, despite an immense success of OFC in both academic and commercial research settings, it has been realised that any OFC technology has a number of drawbacks that originate from fundamental physical limits of light. For example, this is the case in underwater communication [176,177,178] and in some medical imaging and sensing technologies used deeply inside a living human body [35,36], where the intensity of light is significantly attenuated due to scattering and optical absorption in body tissues. This situation has motivated development of alternative approaches exploiting the well-established fact that optical waves share many fundamental physical properties with sound waves [162]. Subsequently, it has been suggested that certain optical precision measurement and sensing technologies, including OFCs, could be implemented using acoustic waves [162].

For a review of the recent developments in the emergent field of acoustic frequency combs (AFCs) readers are referred to [87]. Here, we first describe an important class of AFCs based on the oscillations of acoustically-driven bubbles in liquids and then discuss their potential applications in the field of biosensing.

### 4.1. Physical Principles of Operation of Bubble-Based Acoustic Frequency Combs

One well-established approach to creating OFCs exploits nonlinear optical effects [179] to generate an optical spectrum containing a large number of frequency peaks [180]. Recently, it has been suggested [162] that acoustic nonlinearities [181] can result in the generation of AFCs that are conceptually similar to OFCs. The so-generated AFCs have a significant advantage over its OFC counterparts: while fundamental physical limitations do not allow increasing the intensity of the laser beam indefinitely to amplify nonlinearities [179] and to increase the number of peaks [162], nonlinear acoustic effects are intrinsically very strong [181]. It suffices to use an acoustic wave with a low peak pressure to generate an AFCs suitable for practical applications. As a result, equipment needed to generate an AFC is much simpler than optical setups used to produce OFCs, and the energy it requires is significantly lower than what is needed for an OFC signal [162]. We detail this next in the context of gas bubbles.

When an acoustic pressure wave propagates through a liquid such as water, its initially sinusoidal waveform changes so that its initial monochromatic spectrum acquires higher harmonic frequencies. The more nonlinear the medium in which sound propagates, the stronger such a spectral enrichment [162]. The degree of acoustic nonlinearity is often characterised by the acoustic parameter $\beta =\frac{B}{A}$, which is the ratio of coefficients *B* and *A* of quadratic and linear terms in the Taylor series expansion of the equation of state [181],

$$p=p\left(\rho \right)\approx p_{0}+A\frac{\rho -\rho_{0}}{\rho_{0}}+B\frac{{\left(\rho -\rho_{0}\right)}^{2}}{2\rho_{0}^{2}}+\cdots$$

relating the thermodynamic pressure *p* in the medium with its density ρ (subscript 0 denotes values in the absence of sound). The larger the value of β, the more nonlinear the medium, the stronger a distortion of the acoustic spectrum from the initial monochromatic state [162]. For example, water with β=3.5 is more acoustically nonlinear than air with β≈0.7, but the degree of nonlinearity is moderate in both media. However, when air bubbles are injected into water, the value of β increases to around 5000 and one observes giant acoustic nonlinearities and relevant physical phenomena that are associated with bubble oscillations in liquids [1,2,3,46,62,63,64,67,82,83,97,181,182,183].

**Figure 12:**
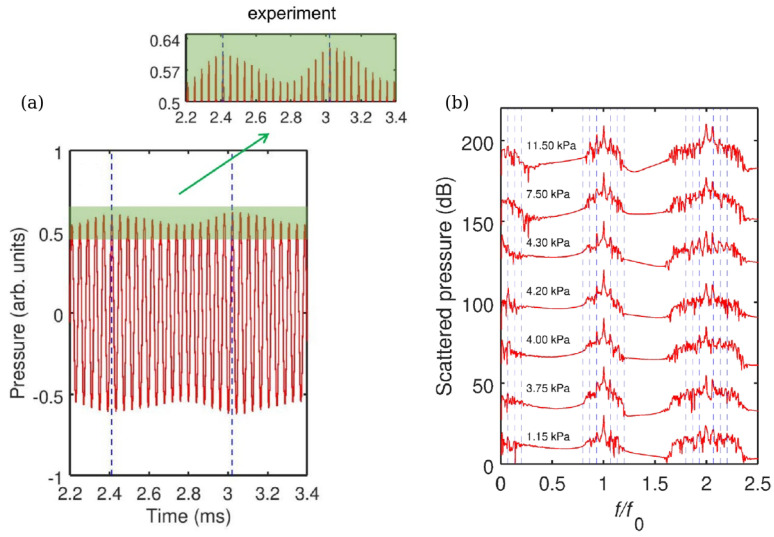
(**a**) Measured acoustic response of a bubble. The time between the vertical dashed lines is $\Delta T=1/f_{nat}\approx 0.6$ ms, where $f_{nat}$ is the natural frequency of the bubble oscillations (see [184] for details). The insets show a closeup of the waveforms and demonstrate the amplitude modulation. (**b**) Experimental AFC spectra obtained using gas bubbles in water insonated with an $f_{0}=24.6$ kHz sinusoidal signal of increasing pressure amplitude α =1.15, 3.75, 4, 4.2, 4.3, 7.5 and 11.5 kPa. The scattered pressure values (in dB) are given along the vertical axis with a vertical offset of 30 dB between the spectra. Reproduced from [184] published by Springer Nature under the terms of the Creative Commons CC BY license.

The idea to employ bubbles to generate an AFC has been validated experimentally in [184], where a single-frequency ($f_{0}=24.6$ kHz) ultrasound wave irradiated several air bubbles created in a water tank using a generator. A small (not exceeding 11.5 kPa) peak pressure amplitude of the driving ultrasound wave was deliberately chosen since, as discussed above, low-amplitude signals suffice to induce strong nonlinearities in liquid–gas mixtures. The generated bubbles had the equilibrium radii $R_{0}\approx 1.0\pm 0.5$ mm. However, since they interacted with each other during the oscillation driven by an ultrasound wave, a collective acoustic response typical of a small bubble cluster with an effective natural frequency $f_{nat}\approx 1.7$ kHz was observed [2,3]. Using high-speed imaging and following [185] it was demonstrated that the resulting cluster behaved similarly to a large single bubble with an equilibrium radius of 1.95 mm. Thus, since $f_{nat}$ is an order of magnitude lower than the frequency of an ultrasound wave, the bubble cluster oscillations resulted in a nonlinear generation of multiple ultraharmonic frequency peaks in the spectrum of the acoustic response. The interaction of the so-generated acoustic waves with the noise-induced bubble oscillations at their natural frequencies resulted in the amplitude modulation of the collective bubble response (Figure 12a) and the appearance of sidebands around the harmonic and ultraharmonic peaks (Figure 12b). These sideband structures can be used as AFCs.

The analysis of experimental results was supported by a rigorous theoretical and computational modelling of bubble oscillations using the RP equation presented in Section 2. It was shown that both experimental time-domain signals and AFC spectra are in good qualitative agreement with the calculated ones. However, because the numerical model based on the RP equation considered only a single oscillating gas bubble with an effective equilibrium radius of 1.95 mm, it was unable to reproduce the generation of another AFC spectrum centred at the second harmonic of the driving ultrasound wave. In fact, Figure 12b shows a sideband peak structure at 49.2 kHz (i.e., $f/f_{0}=2$) and peak ultrasound wave amplitude α=4.3 kPa. As shown in [184], at that frequency the amplitude modulation also gives rise to a train of pulses with the modulation period close to that of the natural bubble cluster oscillations, confirming that this signal can also be used as an AFC. Furthermore, a slight irregularity of the AFC peaks in Figure 12b was attributed to Doppler effect associated with a translational motion of oscillating bubbles in the incident ultrasound field [46]. The size variation of the generated bubbles could also contribute to the comb peak imperfection. However, these deficiencies were not considered prohibitive, which is demonstrated in the following section.

### 4.2. Spectrally Wide Acoustic Frequency Combs

For certain applications the AFC spectrum has to span over an octave of bandwidth (i.e., the highest frequency in the FC spectrum has to be at least twice the lowest frequency). To achieve this, the AFC spectrum can be extended using one of the techniques developed, for example, for broadening spectra of opto-electronic FCs [186] (as demonstrated in [162], the adoption of optical techniques in acoustics is possible because of the analogy between nonlinear optical processes in photonic devices and their counterparts in liquids containing gas bubbles). Furthermore, analysis developed in [184] demonstrated that the number of peaks in a nonlinearly generated AFC and their magnitude can be increased by simultaneously decreasing the frequency and increasing the pressure of the ultrasound wave driving bubble oscillations.

Yet, as seen from Figure 12b the shape of spectral peaks is somewhat irregular. It could be argued that this artefact is associated with a translational motion of oscillating bubbles in the incident ultrasound field. Therefore, the question of long-term stability of AFC signals arises, which was comprehensively discussed in [86] using the model of interacting oscillating bubbles presented in Section 2.

In [86], an alternative strategy for broadening spectra of AFCs generated using bubble oscillations was suggested. The question of long-term stability of AFC signals was comprehensively addressed (see also [87]). In [86], polydisperse clusters consisting of mm-sized bubbles with equilibrium radii $R_{n0}=R_{10}/n$ were used, where $R_{10}$ is the equilibrium radius of the largest bubble in the cluster and n=1,2,3,… is the total number of bubbles. Although clusters with other bubble size distributions could also be used in the proposed approach, it was shown that this specific ratio of equilibrium radii enables generating AFCs with a quasi-continuum of equally spaced peaks. Similarly to the experiment [184], in the analysis performed in [86] low-pressure ultrasound waves (up to 10 kPa) were considered and a numerical model of dynamics of multibubble clusters with translational motion developed in [46,94,103] was employed (see Section 2).

**Figure 13:**
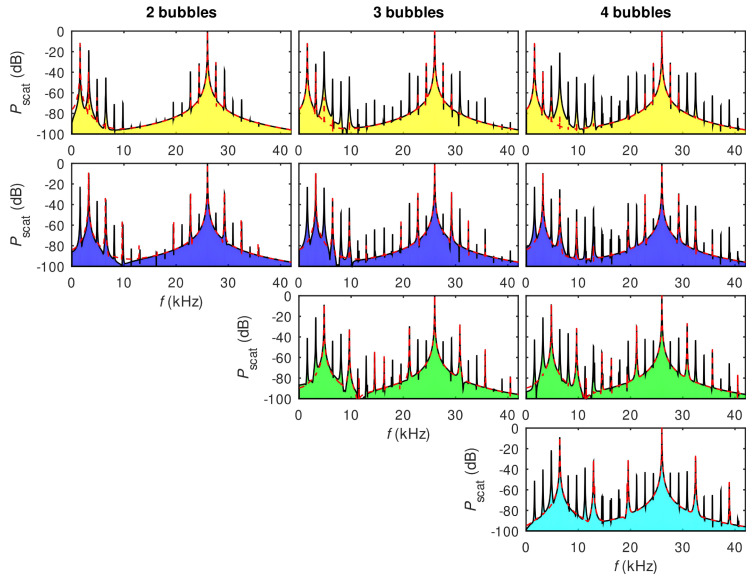
Columns (from left to right) show the AFC spectra produced by individual bubbles within clusters consisting of two, three and four bubbles with the equilibrium radii $R_{n0}=1.95/n$ mm, where *n* is the bubble index in the cluster. The number of panels in each column corresponds to the total number of bubbles. The red dashed lines in each panel show the spectra of individual non-interacting stationary bubbles with identical equilibrium radii. Computational parameters are given in [86]. Reproduced from [86]. Copyright 2021 by the American Physical Society.

Figure 13a shows the calculated spectra for several bubble clusters. The number of rows in each column corresponds to the total number of bubbles in the cluster. Each column shows the spectrum of the pressure scattered by an individual bubble within the cluster. The inspection of panels within the same row from left to right reveals changes in the AFC peak structure caused by the addition of smaller bubbles to the cluster. For example, the four panels in the top row show that the number of equidistant peaks in the AFC spectrum produced by the largest bubble increases when smaller bubbles are added. This is because bubbles within a cluster are affected by the pressure waves scattered by their neighbours and thus their spectra include additional frequency peaks compared to the spectra of isolated non-interacting stationary bubbles of the same equilibrium radii (the dashed lines in Figure 13). Similarly, panels in the second row show the evolution of the AFC spectrum of the second largest bubble in the cluster, and so on. In all cases, the spectra exhibit a key feature of a pure AFC signal—the spectrum of the acoustic response of each bubble consists of a series of well-defined equally spaced peaks.

Finally, to examine the basic trends in the temporal stability of a bubble cluster, a system of two interacting bubbles in liquid was analysed using the model presented in Section 2. In particular, primary (due to acoustic pressure [99,100]) and secondary (arising between two or more interacting bubbles [46]) Bjerknes forces were computed in [86]. It was demonstrated that these forces are small at the typical driving frequencies used in the generation of bubble-based AFCs away from bubble resonances. Therefore, acoustic bubble response can be measured and recorded for AFC applications before bubble oscillations become affected by their aggregation. Numerical calculations carried out using a model that considers more than two interacting bubbles also demonstrated that the frequency of ultrasound wave driving bubble oscillations can be chosen in a wide spectral range above the natural oscillation frequency of bubbles in a cluster. This can greatly facilitate the generation and recording of stable AFC signals since at low pressure bubble clusters exhibit a regular behaviour for a longer time.

### 4.3. Application of Bubble-Based AFCs in Biosensing

**Figure 14:**
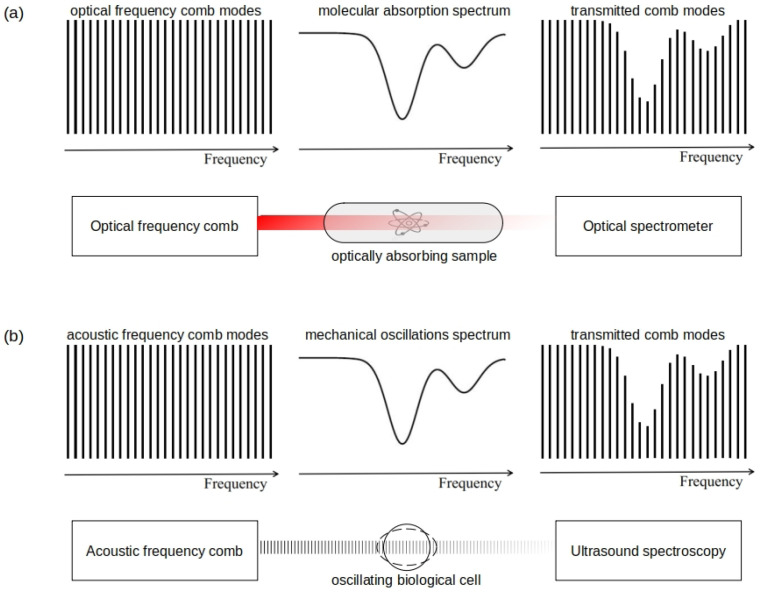
(**a**) Sketch of an OFC-based spectroscopy technique. The OFC as a broadband light source interrogates an absorbing sample and a spectrometer analyses the transmission spectrum revealing the sample’s molecular composition. (**b**) Sketch of a bubble-generated AFC-based spectroscopy technique implemented as an extension of mechanical property measurements of a biological cell illustrated in Figure 10b. AFC as a broadband acoustic pressure wave source interrogates a mechanically oscillating cell and an ultrasound spectrometer analyses the transmission spectrum that provides information about mechanical properties of the cell and its individual organelles.

OFCs are widely used in the field of spectroscopy [87,175,187,188], where they both optically excite and interrogate a sample under study (Figure 14a). The spectral response of a sample, which may arise due to linear or nonlinear absorption of light specific to the sample’s molecular composition, may span the entire OFC spectrum. To conduct measurements existing spectrometers have been adapted and improved to resolve individual OFC peaks.

Similar spectroscopic measurements can be conducted using an AFC signal generated using a cluster of oscillating gas bubbles that are located near a biological cell or bacterium, an arrangement illustrated in Figure 14b and Figure 10b. As with OFC-based spectroscopic measurements, oscillations of a bubble cluster produce an AFC signal that acoustically excites and interrogates the cell. The spectral response of a cell is defined by its mechanical properties. It can be detected using a technically simple high-frequency electronic circuit. Since in this potential experimental scheme a mechanical response of a biological cell is simultaneously detected at several acoustic frequencies, the use of AFC may open opportunities for estimating mechanical properties of different organelles within a living cell while conducting a single measurement. This would be advantageous compared with a series of independent measurements at multiple frequencies because the cell under study moves continuously and inaccuracies associated with such a motion would need to be somehow accounted for if individual measurements are staggered in time.

## 5. Applications of Gas Bubbles in Photoacoustic and Acousto-Optical Biosensors

Research synergies between physicists, chemists, biologists and clinicians have led to the development of novel biomedical imaging and sensing techniques that are less invasive and can provide increased sensitivity and resolution compared with the previous generation technologies. These novel approaches include special modalities of ultrasound and photoacoustic imaging that can also be used to detect mechanical properties of soft biological tissues and, potentially, of individuals cells [35,189,190]. In particular, while it is well-known that ultrasound imaging has several advantages over the competing optical and magnetism-based imaging techniques in terms of resolution, penetration depth, cost effectiveness, imaging system portability and the ability of ultrasound to access hard-to-reach places such as blood vessels in a living body, they can be enhanced even further using artificial ultrasound contrast agents such as gas bubbles [72,73] and nanoparticles that combine magneto-optical and plasmonic properties [36,164].

However, in some practical situations even the most advanced ultrasound imaging systems cannot provide sufficiently high resolution and sensitivity. This is the case, for example, with detecting melanin molecules in tissues [191]. To resolve this problem, ultrasound imaging systems can be combined with photoacoustic in which a pulsed laser beam is used to excite ultrasound pressure waves. Several physical mechanism such as thermal expansion, vaporisation, photochemical processes and optical breakdown [35] underpin their operation. However, in biomedical applications of photoacoustics the only biologically safe mechanism is thermal expansion, where absorption of the laser light by a biological tissue results in the local heating and the generation of broadband ultrasound signals. These signals are detected using piezoelectric transducers that convert them into electric pulses processed to produce an image. Thus, photoacoustic modalities including intravascular photoacoustic imaging [35] inherit a large penetration depth and high spatial resolution characteristics of ultrasound imaging and possess high resolution and sensitivity that are the characteristics of optical imaging systems [36].

It is instructive to note a conceptual difference between photoacoustic and acousto-optical physical processes. Acousto-optics is a branch of physics that studies interactions between acoustic and optical waves [192], for example, the diffraction of a laser light by sound. As another example illustrating an acousto-optical interaction one can think of light being scattered by the areas of a liquid or a biological cell that are subjected to temporal density variations caused by an acoustic pressure wave. This is the case in Brillouin scattering processes (Section 3.1).

Returning to the discussion of photoacoustic imaging we note that thermal expansion—the safest and thus the main physical mechanism underpinning biomedical photoacoustic imaging—is one of the least efficient light-ultrasound interaction processes [35]. A laser light with intensity that is safe for a living organism produces low-amplitude acoustic pressure wave, which limits capabilities of a photoacoustic imaging system. On the other hand, since the contrast in photoacoustic imaging arises from a natural variation in the optical absorption of the tissue components that can be enhanced by using optical contrast agents such as plasmonic nanoantennas [36,164]. While they can play diverse roles depending on their particular application, biomedical photoacoustic imaging systems have been shown to benefit from specially designed nanoantennas with an optical absorption cross-section that is many orders of magnitude larger than that of the targeted biological tissues [193]. For instance, there have been reports of measurements of the impact of plasmonic nanontennas on the identification of macrophage cells playing an important role in atherosclerosis [35,193]. The investigated cells were loaded with 50 nm-diameter spherical gold nanoantennas to demonstrate that, compared with the spectrum of free gold nanoantennas, the spectrum of the nanoantenna-laden was red-shifted and the linewidth of the frequency peak was broadened. These effects are due to a cumulative influence of plasmon resonance coupling of adjacent gold nanoantennas after they are internalised by macrophages. Both these effects can be employed for sensing and imaging purposes [36].

**Figure 15:**
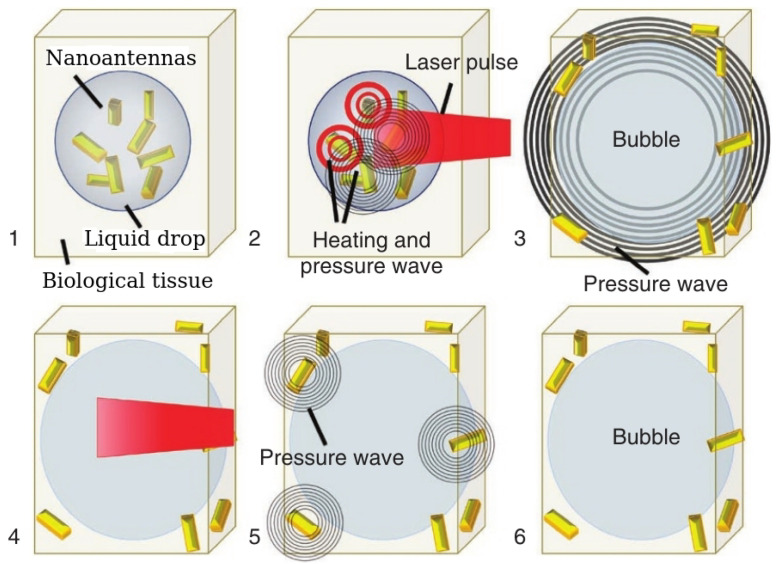
Diagram of a step-by-step remote activation of photoacoustic contrast agents via two physical mechanisms: vaporisation of liquid nanodroplets (steps 2 and 3) and thermal expansion caused by plasmonic nanoantennas (steps 4 and 5). The formed microbubble (step 6) provides contrast for ultrasound imaging. Reproduced from [194] with permission of Springer Nature. Copyright 2012.

Another relevant approach was proposed and experimentally validated in [194], where the process of liquid vaporisation was exploited to generate photoacoustic signals with a significantly larger amplitudes compared with those achievable using traditional physical mechanism of thermal expansion of biological tissues. The novel contrast agent (Figure 15) consists of liquid perfluorocarbon nanodroplets that contain encapsulated plasmonic nanoantennas. As a result of irradiation of such nanodroplets with laser pulses perfluorocarbon undergoes a liquid-to-gas phase transition generating giant photoacoustic transients from ultrasmall nanoantennas. Once the transition to a gaseous phase is completed, it provides ultrasound contrast enhancement due to formed gas bubbles.

**Figure 16:**
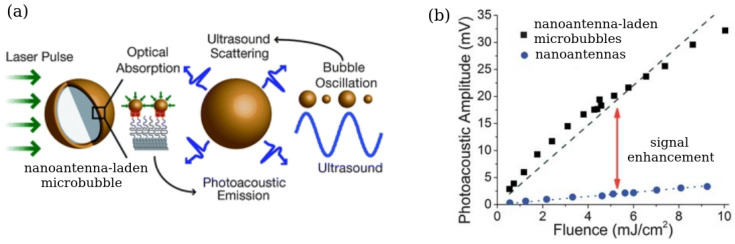
(**a**) Illustration of the operating principles of a nanoantenna-laden microbubble contrast agent. Superior photoacoustic properties are achieved owing to high optical absorption of nanoantennas embedded into the microbubble shell. These ultrasmall nanoantennas do not affect acoustic properties of a microbubble allowing it to serve as an efficient contrast agent for ultrasound waves. (**b**) Amplitude of the photoacoustic response obtained at various fluences of a laser light for the same nanoantenna-laden microbubble contrast agents and free-standing nanoantennas. The vertical double arrow indicates the enhancement of a photoacoustic signal due to the integration of nanoantennas with a microbubble. Reproduced from [195] with permission of Royal Society of Chemistry. Copyright 2013.

A conceptually similar approach was proposed in [195], where nanoantenna-laden microbubbles capable of simultaneous contrast enhancement in both ultrasound and photoacoustic imaging were employed (Figure 16a). The fabrication of such dual-modality contrast agents relies on specific binding of gold nanoparticle antennas to a biotinylated microbubble shell providing stoichiometric control of the nanoantenna surface density and optical absorption while still retaining acoustic properties of a microbubble. Nanoantenna-laden microbubbles were shown to be very efficient photoacoustic agents that have an enhanced response compared to free nanoparticles (Figure 16b).

### Acousto-Optical Sensors Using Bubbles

In this section, we review studies of acousto-optical sensing using gas bubbles in liquids. Our discussion will focus on Mie scattering and bubble-on-fibre acousto-optical devices that hold the potential for applications in biosensing systems discussed in the preceding sections.

In electromagnetism and optical physics the term ‘Mie scattering’ is often used in the context of a solution of Maxwell’s equations that describes scattering of a plane electromagnetic wave by a homogeneous sphere. The solution takes the form of an infinite series of spherical multipole partial waves and is named after its author Gustav Mie [196,197]. For spherical particles that are much smaller than the wavelength of the scattered light, there are simple and accurate approximations of the full solution that adequately describe a particle response. Mie theory also remains valid for objects such as water drops in the atmosphere, biological cells, cellular components and microbubbles that are larger than the wavelength of the scattered light provided that several changes are introduced [198,199]. This extended approach has been used extensively in meteorological optics and it has also become the primary method for determining bubble size in optically transparent liquids [200,201]. Subsequently, Mie theory and the experimental techniques build around it can be used as a means for reading the state of gas bubble-based sensors.

However, in many scenarios a sensor has to operate in hard-to-reach locations within a human body, e.g., inside blood vessels [35]. This means that freely propagating light cannot be used to read its state since body tissues and fluids are mostly impassable to visible and infrared light [202]. Surgical intervention needs to be used to deliver light to an internal body organ, but it has to be carefully planned to minimise risks and to avoid adverse effects and postoperative complications [203].

One strategy to accomplish minimally invasive sensing and imaging inside a living body is to insert an optical fibre, which dramatically simplifies a surgical intervention procedure and minimises a postoperative wound and reduces a chance of its infection [204,205,206]. An optical fibre can be introduced through a small hole in the skin and its presence is well-tolerated by internal organs, for example, the spinal column [207] or the brain [208,209]. A similar approach can be used to sense and image individual biological cells. The benefits of using a miniaturised optical fibre [210,211] to deliver light to a sample include higher spatial resolution and increased sensitivity due to the fibre’s ability to manipulate light, which is impossible when a direct light beam is used.

Furthermore, as with intravascular photoacoustic imaging techniques [35], an optical fibre can be integrated with a miniaturised electric wire that feeds a small transducer used to detect ultrasound waves. Such an engineering solution allows adding another sensor component that can probe mechanical properties of individual biological cells and tissues—a gas bubble (see Section 3). Although such bubble-on-fibre biosensors have not been manufactured yet, several experimental studies demonstrate their plausibility and potential applicability in relevant fields [212,213].

**Figure 17:**
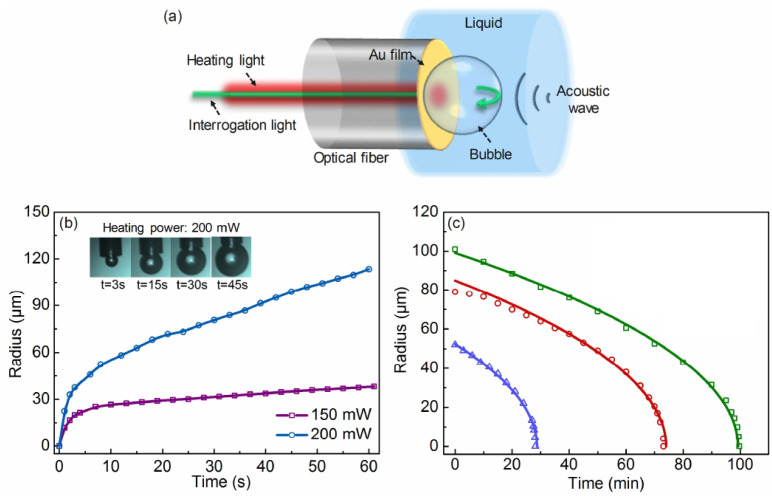
(**a**) Schematic view of a bubble-on-fibre system for detecting acoustic pressure waves. The system consists of a conventional single-mode optical fibre used to deliver both heating and interrogation light beams to a bubble. A thin gold film is used to absorb light and produce Joule heat needed to generate a microbubble. (**b**) Temporal microbubble growth at heating light powers of 150 and 200 mW. The inset shows snapshots of a growing microbubble. (**c**) Bubble decay after the heating light was turned off. The green curve with squares shows the behaviour of a bubble that reached radius of 101 μm. The other two curves correspond to smaller bubbles with radii of 79 μm and 52 μm, respectively. Reproduced from [212]. Copyright 2018 Optical Society of America under the terms of the OSA Open Access Publishing Agreement.

In experiments reported in [212] a tuneable broadband acousto-optical sensor was built using a bubble-on-fibre interferometric approach (Figure 17a). A single microbubble was generated using a heating laser beam at the wavelength of 980 nm. From a physical point of view, the so-created bubble forms a spherical opto-mechanical micro-resonator in water [162], where mechanical deformations of the bubble caused by external acoustic pressure waves change its shape and dramatically alter the optical resonance properties, which was detected using another laser beam at wavelength of 1550 nm. It was shown that at constant power of a heating laser the radius of a generated bubble increases with time (Figure 17b) but it starts decreasing when the heating light is turned off (Figure 17c). Such a bubble behaviour may be used for sensing applications, where a single measurement takes about a second during which the bubble retains the required size. Alternatively, it should be possible to postprocess raw data by removing a background signal that originates from a slow change in the bubble size.

Now let us turn attention to the use of an ultra-thin gold film at the tip of a fibre immersed in water (Figure 17a) [211]. Such a setup can support plasmon resonance underpinning the functionality of many optical sensors [164]. It is also well-known that nanopatterning of thin films results in enhanced plasmonic properties such as the localisation of light at the nanoscale and the increase in light-matter interaction. Combined, they lead to higher sensitivity of a sensor [161,211,214].

**Figure 18:**
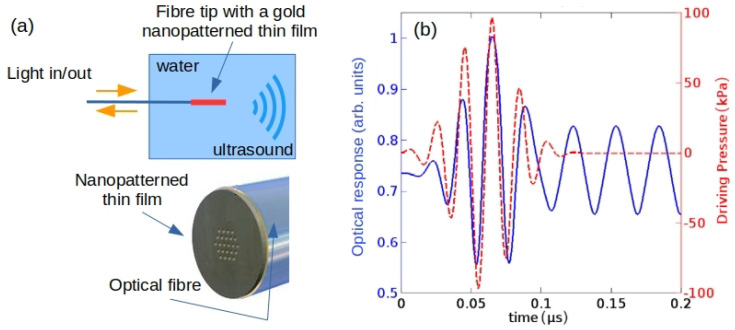
(**a**) Schematic diagram of a bubble-on-fibre system employing an optical fibre with the tip covered by a thin metal film patterned with nanoscale through holes. Such a film can be used (i) to generate a bubble by laser heating, (ii) to create conditions for extraordinary optical transmission (see the main text) and (iii) to host bubbles inside the holes. (**b**) Simulated optical response of a single water-filled hole in a metal film containing a bubble. The intensity of light interacting with the hole-bubble system becomes modulated due to bubble oscillations caused by an ultrasound pressure wave. Reproduced from [218]. Copyright 2017 by the American Physical Society.

Subsequently, it has been suggested that the operation of a bubble-on-fibre system could be facilitated using a metal film with nanoscale through holes (Figure 18a). The role of such a metal film is threefold. Firstly, similar to the uniform thin film in Figure 17a, it can be used to convert the energy of an incident laser beam into Joule heat needed to generate a microbubble. Secondly, thin films with holes are known to posses unique optical properties such as an extraordinary optical transmission that we will discuss in detail below. Thirdly, if the holes are sufficiently wide and deep, they can be filled with water and host bubbles.

The effect of extraordinary optical transmission is typically observed in a single circular hole in an opaque metal film, when its diameter *w* is much smaller than the wavelength $\lambda_{0}$ of an incident light. Under such conditions, the classical aperture theories [215,216] become invalid since, in contrast to their predictions, the intensity of light transmitted through the hole increases. In general, this effect is achievable in both single and periodically arrayed holes. It is due to the light interaction with plasmon resonances [164] at the surface of the metal film and Fabry–Perot resonances of guided optical modes inside the hole [215,216]. The latter give rise to a frequency peak in the spectrum of the light transmitted through a single hole when $\lambda_{0}\approx \lambda_{c}$, where $\lambda_{c}\propto wn_{f}$ is the cutoff wavelength of the fundamental guided optical mode in an infinitely deep hole of the same size and $n_{f}$ is the refractive index of the material filling the hole. Thus, the frequency of the maximum optical transmission through the hole becomes spectrally tuneable by either changing *w* or controlling $n_{f}$. Other types of electromagnetic waves, e.g., microwaves, may also be transmitted through subwavelength apertures in a fashion similar to light, and enhanced transmission of sound through acoustically subwavelength apertures has also been observed [217].

Of course, reflected light (and waves of other nature) also becomes modified enabling one to observe the extraordinary wave behaviour both in the transmission and reflection modes. Subsequently, an improved bubble-on-fibre system can be proposed (Figure 18a), where a fibre with a tip that is integrated with an array of nanoscale holes is used to both create a microbubble and to detect its oscillations. Alternatively, if holes are sufficiently large, bubbles may be trapped inside them. In either case, due to bubble oscillations the effective optical refractive index $n_{f}$ of the medium that is perceived by the light becomes modulated: an expanding or shrinking bubble occupies respectively a larger or smaller volume creating a larger or smaller region with the refractive index $n_{f}=1$ (air) or $n_{f}=1.33$ (water).

The behaviour of a small bubble trapped inside a hole was analysed in [218] using a model based on the RP equation for a single bubble oscillating in water (Section 2) and extended using O$\tilde{g}$uz-Prosperetti theory developed to predict the frequency of natural oscillations of a spherical bubble placed inside a water-filled rigid circular tube [219]. An integration of the result with that of a numerical model of light propagation inside a hole based on Maxwell’s equations [218] demonstrated that the intensity of transmitted and reflected light becomes modulated by the bubble oscillations (Figure 18b). Modelling predicted that light intensity modulation continued after the ultrasound driving bubble oscillation was turned off. However, this effect is rather artificial since it originates from an idealised model. In reality, the bubble oscillation decay is much faster than that predicted by the model [218]. We note that the model used in [218] also considered viscous and thermal boundary layers [220] at the hole walls. It was shown that their presence does not qualitatively affect results obtained in a lossless approximation.

**Figure 19:**
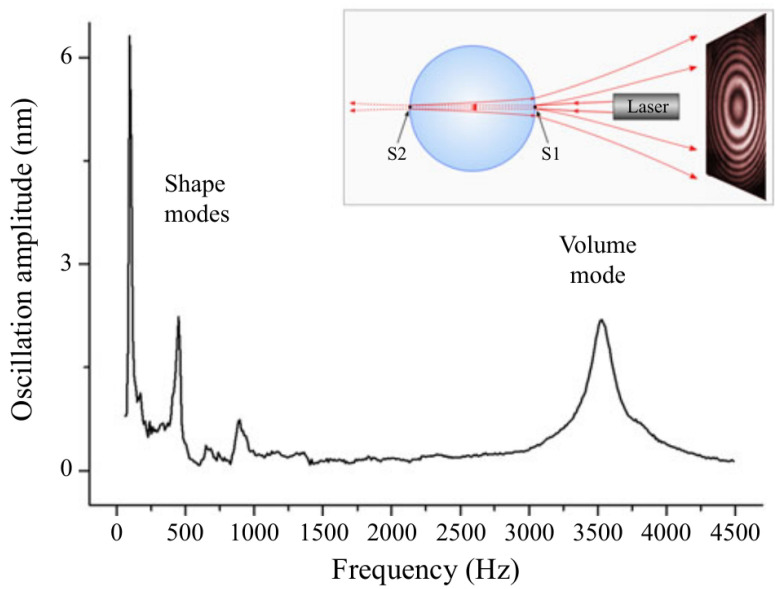
Frequency spectrum of a single bubble with a 0.85 mm radius in water obtained using an optical interferometry technique. The spectrum reveals the presence of (non-spherical) shape oscillation modes in the frequency range from approximately 100 Hz to 1.5 kHz and of a volume oscillation (spherical) mode at approximately 3.55 kHz. The inset shows schematically the detection principle, where a laser beam focused on a bubble undergoes reflections at the curved interfaces (points S1 and S2) generating an interference pattern in the direction opposite to that of the incident laser beam. Reproduced with permission from [221]. Copyright 2022 Cambridge University Press.

Finally, in this section we review an acousto-optical sensing mechanism that exploits gas bubbles and employs an interferometric optical technique to detect the changes in their shape and to investigate interactions between two bubbles undergoing small-amplitude oscillations [221]. This sensing approach relies on the ability of air–liquid interfaces to reflect light similarly to mirrors of a Fabry–Perot interferometer. This effect originates from a difference in the optical refractive indices of the two media. When a laser beam is focused on an oscillating bubble, an optical interference pattern is formed (see the inset in Figure 19), where the optical path length inside a bubble is sensitive to even sub-nanometric deviations from the equilibrium radius [222]. As shown in the main panel of Figure 19, a discrete set of oscillation modes are excited inside the bubble. The peaks on the left and right sides of the frequency spectrum correspond to the (non-spherical) shape oscillations (similar to those of oscillating liquid drops in Figure 9, see [223] for more details) and to the volume oscillation (spherical mode) given by the RP equation in Section 2, respectively. The same interferometric optical technique has also been applied to study the behaviour of individual bubbles [222]. In the two-bubble case it provides an opportunity to directly measure the relative bubble oscillation phase, which could be used to improve performance of AFC-based sensors described in Section 4.

## 6. Gas Bubble Sensors and Artificial Intelligence Algorithms

In this section, we discuss the possibility of using artificial intelligence (AI) algorithms to improve performance of bubble-based sensors discussed in the previous sections. To support this discussion, we review relevant recent studies [224,225] focused on AI-based forecast of the behaviour of bubbles oscillating near an elastic boundary. Readers wishing to familiarise themselves with fundamentals of artificial neural networks discussed in this section may refer to [226].

Traditionally, scientific research has focused on developing theories, refining them using data generated in experiments and analysing those data for creating new technologies. However, with the current fast-paced advances in the field of AI and the burgeoning amount of scientific data, novel data-driven approaches to scientific research have become popular. In particular, in some fields the reliance on existing well-defined theories has diminished in favour of machine learning (ML) algorithms that can be implemented to analyse a problem using only the available data. However, despite certain progress in this direction, an AI-based approach to scientific research often fails when dealing with highly nonlinear systems including living organisms and financial market [227]. This is because to analyse such systems ML algorithms have to rely on big data sets generated during long experimental observations demanding substantial computational resources to make practically useful predictions. Moreover, while pure data-driven models can fit observational data with a reasonable accuracy, their predictions may not always be physically consistent and reliable due to certain observational biases. Therefore, there is an urgent need for integrating fundamental physical laws into the data-driven approaches by augmenting ML models with physical governing equations that guarantee strong theoretical backing of the used algorithms [228].

To implement this new approach to ML, a family of physics-informed neural networks (PINNs) have been introduced, where a ML algorithm combines data with mathematical models such as partial differential equations (PDEs) [229]. Such a PINN can yield more interpretable ML models that remain robust in the presence of imperfect data (e.g. missing or noisy values), which is often encountered in biosensing, and provide accurate and physically consistent predictions [229]. It is conceivable that the use of PINNs will improve performance of different classes of sensors including those based on bubble oscillations. Therefore, such models can be integrated with the existing ML algorithms as demonstrated, for example, in [230].

In a generic neural network, the loss function such as the mean squared error (MSE) quantifies the difference between the expected real-life outcome and that produced by a ML model. From the loss function one can derive feedback to be used for updating properties of a neural network. In line with this approach, PINN integrates information collected from measurements and relevant mathematical models by embedding known PDEs into the loss function of a neural network [231].

**Figure 20:**
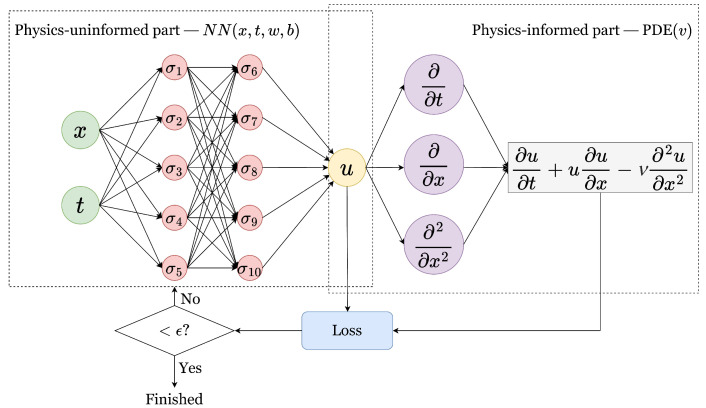
Schematic of PINN for solving forward problems with conventional (the left dotted box) and physics-informed (the right dotted box) neural networks with trainable weights *w* and biases *b* and a nonlinear activation function σ. PINNs integrate the measured and observed information with that produced using physical models by embedding the corresponding PDEs into the loss function of a neural network at a training stage. A set of sampled inputs $\left(x_{i},t_{i}\right)$ is passed through the network, then the Jacobian of the neural network’s output is computed with respect to these inputs, and finally, the residual of PDEs is computed and added as an extra term in the loss function. The network is trained by minimising the loss via a gradient-based optimisation method until it is smaller than a set threshold ϵ.

Figure 20 shows a schematic of the PINN algorithm for solving a forward problem (see [226] for details) using viscous Burger’s equation arising in the fields of nonlinear acoustics and gas dynamics and often employed in problems concerned with nonlinear dynamics of oscillating bubbles [162,214]. In a one-dimensional space, for a given field u(x,t) and the kinematic viscosity ν Burger’s equation reads [232]:

(35) $$\frac{\partial u}{\partial t}+u\frac{\partial u}{\partial x}=\nu \frac{\partial^{2}u}{\partial x^{2}}\;.$$

In Figure 20, a conventional (physics-uninformed) neural network relies on the physics-informed solution u(x,t) of a PDE. The loss function of PINN contains two terms that use data from neural network measurements of *u* based on the initial and boundary conditions and data that is sourced from PDE:

$$L=w_{data}L_{data}+w_{PDE}L_{PDE}\;,$$

where

$$\begin{array}{ccc} L_{data} & = & \frac{1}{N_{0}}\sum_{i=1}^{N_{0}}{\left(u\left(x_{0}^{i},t_{0}\right)-u_{0}^{i}\right)}^{2}+\frac{1}{N_{b}}\sum_{i=1}^{N_{b}}{\left(u\left(x_{b}^{i},t_{b}\right)-u_{b}^{i}\right)}^{2}\;\mathrm{and}\;\\ L_{PDE} & = & {\left.\frac{1}{N_{PDE}}\sum_{j=1}^{N_{PDE}}{\left(\frac{\partial u}{\partial t}+u\frac{\partial u}{\partial x}-\nu \frac{\partial^{2}u}{\partial x^{2}}\right)}^{2}\right|}_{\left(x_{j},t_{j}\right)}\;. \end{array}$$

Here ${\left(x_{0}^{i},t_{0}\right)}_{i=1}^{N_{0}}$ and ${\left(x_{b}^{i},t_{b}\right)}_{i=1}^{N_{b}}$ are two sets of collocation points denoting the initial and boundary locations, respectively, and ${\left(x_{j},t_{j}\right)}_{j=1}^{N_{PDE}}$ represent the collocation points in the entire domain. Additionally, $w_{data}$ and $w_{PDE}$ are the weights used to balance the interplay between the two loss terms. These weights play an important role in improving the trainability of PINN. They can be user-defined or tuned automatically. The network is trained by minimising the loss via a gradient-based optimisation method until the loss is smaller than a set threshold ϵ. Using this PINN scheme, it was demonstrated in [229] that the solution predicted by the neural network coincided with an exact solution of Equation (35) within graphics resolution.

We note that different kinds of PDEs can be used in the PINN scheme outlined above including Shrödinger, Navier–Stokes and Korteweg-de Vries equations [229]. Moreover, in a recent work [230] it has been suggested that the RP equation describing the dynamics of a cluster of bubbles oscillating in a bulk of water can replace a mathematical model underpinning an artificial neural network. Although the relevance of that result to the concept of physically-informed neural networks was not discussed in [230], it is plausible that a network based on the RP equation could produce forecasts that are correct from the point of view of the physics of oscillating bubbles. Although this approach has not been applied in sensing so far, there have been several relevant studies, where the dynamics of oscillating bubbles was predicted using PINN [224,225,233,234]. In particular, in [224] bubble dynamics near various elastic boundaries were predicted using a semi-physics-informed approach based on the fundamental concept of Kelvin impulse [235,236]. This result appears to be of immediate relevance to the discussion in this review article. Similarly, in [225] the microbubble behaviour in microchannel flows commonly encountered in biological applications was predicted using a PINN-like model based on Navier–Stokes equations.

We also foresee a possibility of using a PINN-like approach in the context of bubble-based sensors illustrated in Figure 2, where a PINN algorithm can be employed to forecast the oscillation frequency of a bubble attached to an elastic wall with unknown elastic properties or when a functionalised bubble captures an unknown amount of antigens. In both cases, forecasts can be made using a set of time series data obtained by measuring the bubble frequency near a wall with known elastic properties or with a known amount of binding antigen.

## 7. Bubble Generation

Before we conclude the mainstream discussion, we review the methods of bubble generation. The ability to produce bubbles with required characteristics on demand is important for many applications discussed in this article. The frequency of bubble oscillations depends on their size and shape as well as on their distribution within a cluster. In many laboratory experiments involving individual bubbles (e.g., studies of single-bubble sonoluminescence) a bubble can be created either using a laboratory pipette after adding a small drop of glycerol, which diffuses isotropically in pure water and stabilises a bubble [237], or by local heating of water with a short section of current-carrying Ni-Cr wire [2,70]. Alternatively, an isolated bubble can be created using a short laser pulse [2].

Technically simple equipment, such as an air pump connected to a tube immersed in liquid, is typically used to generate a cluster of bubbles. Depending on the configuration of an experimental setup, the tube may have a series of equidistant holes on its side, the size of which defines the equilibrium radii of the resulting bubbles. In a variation of this setup a piece of porous material such as wood or rubber can be attached to the end of the tube [184]. In this case, the bubble size is controlled by the porosity of the used material and by adjusting the output pump pressure.

However, the aforementioned methods have a number disadvantages including difficulty with the accurate control of the bubble size and of the total number of the generated bubbles. The bubble production rate may also be too slow or too fast for a particular application. Many of these challenges were addressed in studies dealing with bubbles produced in microfluidic systems [238] and used as ultrasound contrast agents [72,73] and as elements of sensors and actuators [17]. For example, in microfludic systems the number and arrangement of the formed bubbles is determined by a pit pattern created on a solid substrate on which the bubbles nucleate. This way the interaction strength between the so-created bubbles is accurately controlled [239]. A similar bubble generation technique is used in acousto-optical devices [218], where microscopically patterned metallic films play the role of a bubble-generating substrate.

In many electrochemical and photo-electrochemical processes individual H${}_{2}$, N${}_{2}$ and O${}_{2}$ bubbles are generated as a result of electrocatalytic reactions on the surface of disc electrodes immersed in an aqueous solution of sulfuric acid [240]. Bubbles can also be electrogenerated in nonaqueous media such as methanol, ethanol, ethylene glycol, acetone and other solvents [240]. An extended discussion of these methods can be found in [241].

Therefore, multiple bubble generation techniques exist that enable a practical implementation of ideas and concepts reviewed in this article. Each method has its own advantages and disadvantages in particular applications, which is often established via trial-and-error testing. However, microbubbles created as ultrasound contrast agents appear to be the main choice in many experiments that are, in general, unrelated to medical imaging [242,243]. The reasons for this are manifold. Firstly, such bubbles can be easily generated in various sizes. Secondly, acoustic and elastic properties of the contrast-agent type microbubbles are well-understood and documented. Finally, an improved mechanical stability and size monodispersity of microbubbles produced in commercial settings enable one to investigate their dynamics at acoustic pressure levels that significantly exceed those encountered in medical ultrasound imaging procedures. For example, contrast-agent type microbubbles were used to demonstrate sonoluminescence in an experiment employing high-intensity ultrasound waves [244]. Such bubbles and methods of their generation are of immediate relevance to the applications discussed in this article.

## 8. Conclusions and Outlook

In this review article, we presented an emergent approach to biomechanical sensing, where a gas bubble oscillating in a liquid is used as the key technological building block. An acoustic pressure wave with the frequency and peak amplitude that are similar to those of ultrasound waves used in medical ultrasound procedures drives bubble oscillations remotely enabling to read its response non-invasively even if it is positioned in a hard-to-reach location. The ability of ultrasound to propagate through biological tissues without damaging them enables a bubble-based sensor to safely probe mechanical properties of cells. This makes such sensing a method of choice compared with fluorescence and atomic force microscopy approaches given that they can damage living cells optically, chemically or mechanically. In addition, encapsulating bubble with bioreceptors enables biochemical functionality of bubble-based sensors.

While development of the concept of bubble-based sensing requires further research and clinical trials, this process is greatly facilitated by the recent progress in adjacent fields of bubble-based ultrasound contrast agents and drug delivery. These technologies have already been commercialised and can serve as precursors for a faster adoption of bubble-based biosensing. Despite that, the physics of bubble dynamics is very rich and its studies are very broad. Therefore, the goal of this review was to bridge the existing gap between theory, proof-of-principle experiments and practical applications. Even though we discussed several conceptually diverse approaches to bubble-based biosensing, no review can encompass all of them. Below we list a few aspects that might be relevant to biomechanical sensing but which are left outside the scope of the current manuscript.

Firstly, collapsing bubbles can emit light in a very wide optical spectral range via the process called sonoluminescence [69,70,237]. The light emission is accompanied by chemical reactions, which are the subject of sonochemistry [19,238]. Although the intensity of sonoluminescence is rather low compared to that used in the well-established optical sensing methods such as fluorescence spectroscopy, it has been argued that the light emitted by collapsing bubbles can be exploited to non-invasively photo-activate drugs inside a living body or a biological cell [244]. Moreover, recent studies [33,240,241] proposed several novel approaches to further enhance sonoluminescence light intensity to a level detectable by standard equipment. In particular, the presence of nanoparticles, which does not affect the acoustic properties of a bubble, amplifies sonoluminescence by fluorescence-like [240,241] and plasmonic [33] effects.

Secondly, bubble clusters with controlled inter-bubble spacings can be generated using light [242] and acoustic pressure waves [243]. For instance, in [243] the secondary Bjerknes force discussed in the main text of the current review has been amplified to enable acoustic assembly and manipulation of centimetre-scale objects. This report presents an interesting opportunity to use bubbles for manipulating individual biological cells or even small pieces of biological tissues and to study their mechanical properties at the same time.

Thirdly, it is known that the surface of oscillating bubbles can exhibit wall ‘shimmering’ [244,245] that is visually detectable surface Faraday waves [246]. The physical properties of Faraday waves also present significant interest for biosensing applications. Interested readers are referred to a recent review [87] that discusses this topic in more detail.

Finally, we mention a possibility of utilising bubble-based sensors in bioengineering for detecting pathogenic organisms [33,247] and creating new kinds of sensors and actuators [248]. In particular, bubbles play an important role in biosensors and actuators based on Marimo, green algae balls *Aegagropila linnaei* that are large colonies of live photosynthetic filaments formed by rolling water currents in freshwater lakes. Photosynthesis in Marimo produces bubbles that attach to its body, changing its buoyancy. This enables Marimo to float in the presence of light and sink in its absence. Apart from being useful in biosensing, the natural motion of Marimo can be used to make oscillators and logic gates [248]. Subsequently, the irradiation of Marimo’s bubbles with acoustic pressure waves may add an extra degree of freedom to controlling its motion. This effect can be exploited to tune the oscillations observed in [248] and to read the state of logic gates proposed there.

## Abbreviations

The following abbreviations are used in this manuscript:

atomic force microscopy	AFM
acoustic frequency comb	AFC
artificial intelligence	AI
blood-brain barrier	BBB
Brillouin light scattering	BLS
in vitro fertilisation	IVF
Keller-Miksis	KM
mean squared error	MSE
machine learning	ML
optical frequency comb	OFC
partial differential equation	PDE
physics-informed neural network	PINN
Rayleigh-Plesset	RP
scanning acoustic microscopy	SAM
signal-to-noise	SNR
